# Impact of sodium‒glucose cotransporter‐2 inhibitors in patients with recent versus previous myocardial infarction: a systematic review and meta-analysis

**DOI:** 10.1186/s12933-024-02540-4

**Published:** 2025-02-13

**Authors:** Pedro Gabriel Scardini, Eric Shih Katsuyama, Alonzo Armani Prata, Julia Marques Fernandes, Christian Ken Fukunaga, Wilson Falco Neto, Ana Carolina Covre Coan, Naieli Machado de Andrade, Abraão Santana Silva, Rafael Petri Pinheiro, Luciana Gioli Pereira, Remo H. M. Furtado

**Affiliations:** 1 Higher School of Sciences of the Holy House of Mercy of Vitória, Av. Nossa Sra. da Penha, 2190 - Santa Luíza, Vitória, ES 29045-402 Brazil; 2 Department of Medicine, FMABC University Centre, São Paulo, Brazil; 3 Federal University of Espírito Santo, Vitória, Brazil; 4 Faculdade Israelita de Ciências da Saúde Albert Einstein, São Paulo, Brazil; 5 FAMECA University Center, Catanduva, São Paulo Brazil; 6 EBMSP, Bahiana School of Medicine and Public Health, Salvador, Brazil; 7 University of Rio Verde, Brasília, Brazil; 8 Federal University of Rio de Janeiro, UFRJ, Rio de Janeiro, Brazil; 9 Brazilian Clinical Research Institute, Sao Paulo, Brazil; 10 Instituto Do Coracao (Incor), Hospital das Clinicas HCFMUSP, Faculdade de Medicina, Universidade de Sao Paulo, Sao Paulo, Brazil

**Keywords:** Sodium‒glucose cotransporter 2 inhibitors, Myocardial infarction, Cardiovascular risk, Systematic review and meta-analysis

## Abstract

**Background:**

Sodium‒glucose cotransporter 2 (SGLT2) inhibitors have been included in heart failure (HF) guidelines because of their benefits in reducing mortality and hospitalization rates. However, the timing and benefits of initiating SGLT2 inhibitors in patients after myocardial infarction (MI) remain controversial. Therefore, we aimed to perform a systematic review and meta-analysis comparing SGLT2 inhibitors with placebo in patients with MI.

**Methods:**

We performed a systematic review and meta-analysis to determine the impact of SGLT2 inhibitors in patients with recent or previous MI. We systematically searched PubMed, Cochrane, and Embase for RCTs comparing SGLT2 inhibitors versus placebo in patients with MI. The primary outcome was (1) HF hospitalization. In this analysis, we also included the following secondary outcomes: (2) major adverse cardiovascular events (MACE) defined as a composite of cardiovascular (CV) death, MI or stroke; and (3) all-cause mortality. A subgroup analysis was conducted for the primary outcome, comparing patients who had experienced an MI more than 8 weeks prior to study enrolment (previous MI) versus those who had experienced an MI within the preceding 8 weeks (acute MI). Risk ratios (RRs) and 95% confidence intervals (CIs) were pooled with a random effects model.

**Results:**

Our meta-analysis included 10 RCTs comprising 22,266 patients, of whom 11,339 (51.2%) had type 2 diabetes. The mean age was 62 years, and the median follow-up was 21 months. According to the pooled analysis, HF hospitalization rates were lower in patients on SGLT2 inhibitors compared with placebo (RR 0.77; 95% CI 0.69, 0.85; *p* < 0.001)). Differences in MACE were also observed in favor of SGLT2 inhibitors versus placebo (RR 0.88; 95% CI 0.79, 0.97; *p* = 0.012). There was no statistically significant difference in all-cause mortality between the groups (RR 0.88; 95% CI 0.78, 1.00; *p* = 0.058). Benefits of SGLT2 inhibitors for the primary outcome were consistent regardless of the timing of last MI, with no treatment by subgroup interaction (p for interaction = 0.56).

**Conclusion:**

In this meta-analysis of patients who experienced MI, the administration of SGLT2 inhibitors was associated with lower rates of hospitalization for HF. In addition, the treatment effect of SGLT2 inhibitors was consistent regardless of whether they were started in the recent versus previous MI setting.

**Supplementary Information:**

The online version contains supplementary material available at 10.1186/s12933-024-02540-4.

## Introduction

Myocardial infarction (MI) remains the main cause of death in the world [1]. The prognosis of MI has improved [2] due to the implementation of early reperfusion, effective pharmacological therapy, evidence-based management of complications, and individualized treatment for specific populations. Despite these advances, there has been a slowdown in improvements in more recent years, with limited new treatment options and a persistent high residual risk of cardiovascular (CV) events after MI [3].

Sodium–glucose cotransporter-2 (SGLT2) inhibitors have emerged as new and widely studied drugs in cardiology because of their positive effects in a wide spectrum of CV and metabolic parameters Recently, SGLT2 inhibitors have been recommended in heart failure (HF) guidelines [4] to reduce cardiovascular mortality and hospitalization caused by HF exacerbations. Additionally, they have proven benefit in patients with chronic kidney disease and type 2 diabetes (T2DM), with or at risk of atherosclerotic CV disease, based on several randomized controlled trials (RCTs) and meta-analyses [5–7].

Previous meta-analyses [8, 9] of patients with acute MI demonstrated a reduction in HF hospitalization with SGLT2 inhibitors but no statistically significant decrease in the other CV outcomes. However, large RCTs testing SGLT2 inhibitors have included subgroup analyses of patients with a history of MI, which warrants further exploration. Moreover, the optimal timing for initiating SGLT2 inhibitors in MI patients remains controversial. Therefore, we aimed to conduct a systematic review and meta-analysis to reassess the efficacy of SGLT2 inhibitors in patients with MI and compare the outcomes between patients with recent versus previous MI.

## Methods

This systematic review and meta-analysis was performed and reported according to the Preferred Reporting Items for Systematic Reviews and Meta-Analysis (PRISMA) Statement guidelines and the Cochrane Collaboration Handbook for Systematic Reviews of Interventions guidelines [10, 11] The prospective meta-analysis protocol was uploaded to the International Prospective Register of Systematic Reviews (PROSPERO; CRD42024566070).

### Eligibility criteria

There were no restrictions in publication date, status, or language. The inclusion criteria were as follows: (1) RCTs comprising patients with MI, either with acute MI diagnosis or a history of previous MI; (2) comparing SGLT2 inhibitors with placebo; and (3) reporting any prespecified efficacy and safety outcomes. Given that SGLT2 inhibitors seem to provide CV benefits regardless of history of MI [7], including both recent and previous MI patients enhances the applicability of our meta-analysis. We excluded studies that did not report any of the outcomes of interest or that had overlapping patient populations. RCTs of SGLT2 inhibitors versus placebo in a general population, including those with or without T2DM, were included only if they specifically reported outcomes for a subgroup of patients with MI.

### Search strategy and data extraction

We systematically searched PubMed/Medline, EMBASE, and Cochrane from database inception to June 2024. The study selection process included an initial review of titles and abstracts, followed by a thorough examination of the full texts of potentially suitable studies. The full search strategy is reported in Supplementary Methods 3. Eight authors (C.F.; N.A.; R.P.; A.P.; W.F.; A.C.; J.F.; A.S.), in pairs, independently and following a double-blinded model, extracted selected studies, reviewed the main reports and supplementary materials and extracted the relevant information from the included trials. Any discrepancies were resolved by consensus among the authors or with deliberation with other review team members (P.S.; E.K.).

### Endpoints and subgroup analysis

The primary endpoint of this meta-analysis was (1) hospitalizations for HF. We also included the following secondary endpoints: (2) all-cause mortality; (3) CV death; (4) major adverse cardiovascular events (MACE), defined as the composite of CV death, MI or stroke; (5) MI recurrence; and (6) stroke. Additionally, we conducted a prespecified subgroup analysis for the primary endpoint, focusing on the effects of the following factors: presence of T2DM, timing of MI (recent vs. previous), and type of SGLT2 inhibitor used (empagliflozin or dapagliflozin). A recent MI was defined as patients having experienced an MI less than 8 weeks prior to either hospitalization or study enrollment. In contrast, patients who had experienced an MI at least 8 weeks before their current hospitalization or study inclusion were classified as having a previous MI. We chose this time frame based on previous literature suggesting that changes in left ventricular volume and function following an MI are typically observed after 8 weeks [12]. Accordingly, we consider it clinically relevant to explore the potential effects of SGLT2 inhibitors both before and after post-MI cardiac remodeling may have occurred. Detailed definitions of the endpoints can be found in Supplementary Methods 4. We performed post hoc sensitivity analysis for the primary outcome stratified by left ventricular ejection fraction (LVEF) < 50% and MI presentation on electrocardiogram, that is, ST-elevation myocardial infarction (STEMI). We also reassessed the primary outcome after excluding trials that were sub-analyses or post hoc in nature.

#### Quality assessment

Eight authors (C.F.; N.A.; R.P.; A.P.; W.F.; A.C.; J.F.; A.S.), in pairs, independently assessed the risk of bias for each trial using the criteria outlined in the *Cochrane Handbook for Systematic Reviews of Interventions *[11] through the Revised Cochrane risk of bias tool for randomized trials (RoB-2) [13]. Disagreements were resolved by consensus or, if necessary, by consulting a third author (E.K.; P.S.). We assessed the risk of bias according to the following domains: random sequence generation, allocation concealment, blinding of participants and personnel, blinding of outcome assessment, incomplete outcome data, selective outcome reporting, and other biases. We graded each trial as having a high, low, or unclear risk of bias for each domain. We also performed funnel plot analysis and the Egger test to assess publication bias [14].

### Statistical analysis

Endpoints were analyzed using a risk ratio (RR) with 95% confidence intervals (CIs). We also computed the hazard ratio (HR) with 95% CIs for a time-to-event sensitivity analysis. We assessed heterogeneity via the Cochrane Q statistic and Higgins and Thompson’s I^2^ using a restricted maximum likelihood estimator. Heterogeneity was low if *I*^2^ = 25%, moderate if *I*^2^ = 50%, or high if I^2^ = 75%. The random effects model was used once we assumed different effect sizes in the selected population. Our prespecified subgroup interaction was performed using the Q test method, following a null hypothesis of no interaction between groups expressed as a p value. The reported p values are two-sided, and we made no adjustments for multiple testing. We performed statistical analyses using R version 4.3.2 (R Core Team, Vienna, Austria) and the R package meta [15].

### Trial sequential analysis

We used TSA 0.9.5.10 Beta software for trial sequential analysis (TSA) to confirm our meta-analysis results. The type of boundary value for the hypothesis test was set to a two-sided test with an alpha value of 5%. Once the cumulative studies in the Z curve cross the conventional monitoring boundary or the futility area, the results are consistent and should be considered reliable evidence [16].

## Results

### Study selection and baseline characteristics

**Fig. 1:**
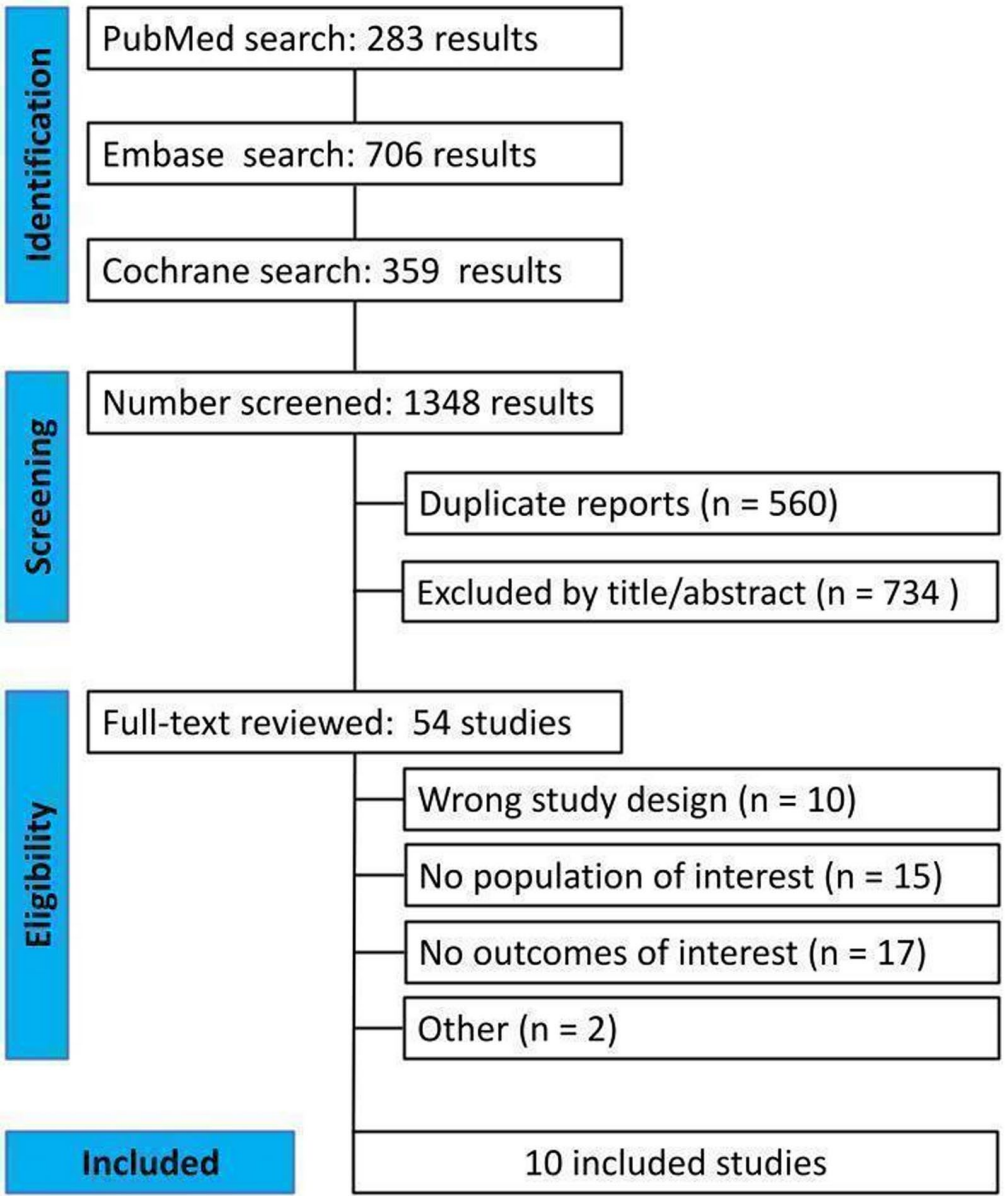
PRISMA flow diagram. Legend: PRISMA flow diagram of study screening and selection

The study selection process is presented in Fig. 1. The initial search identified 1348 studies (PubMed [n = 283], Embase [n = 706], and Cochrane [n = 359]). After title and abstract screening and removal of duplicates, 54 studies remained to be fully reviewed according to the inclusion and exclusion criteria. From these, ten double-blinded, multicenter RCTs and their respective reports were included [17–29] enrolling a total of 22,266 patients, of whom 11,669 (52.4%) were randomized to SGLT2 inhibitors. A full description of the eligibility criteria per study can be found in the Supplementary Table 1. The included participants had a mean age of 62 years, were mostly male (67.4%), and 51.2% had T2DM. The follow-up ranged from 2.8 to 50.4 months, with a median of 21 months. Regarding the intervention, 1 study used canagliflozin (100 mg; 300 mg), 4 used dapagliflozin (10 mg), and the remaining five studies used empagliflozin (10 mg; 25 mg). Table 1 and Supplementary Table 2 present other important characteristics from each study.

**Table 1:** Baseline patient and study characteristics

Study and year	Sample size	SGLT2 inhibitor	Black (n)	Age (y)^†^	Male, n (%)	DM II, n (%)	eGFR (mL/min /1.73 m^2^)^†^	STEMI, n (%)	Follow-up (months)
DAPA MI 2023 [17]	4017	Dapagliflozin 10 mg	23(0.6)	62.9	3210 (79.9)	0 (0)	83.4	2893 (72)	11.6
Adel et al. 2022 [18]	93	Empagliflozin 10 mg	N/A	56	56 (60.2)	93 (100)	N/A	50 (53.7)	6
EMBODY 2020 [19]	96	Empagliflozin 10 mg	N/A	64.4	77 (80.2)	96 (100)	65.4	N/A	5.5
EMMY 2022 [20]	476	Empagliflozin 10 mg	N/A	57	392 (82)	63 (13)	92.0	N/A	6.5
EMPACT-MI 2024 [21]	6522	Empagliflozin 10 mg	92 (1.4)	63.6	4897 (75)	2081 (31.9)	77.8	4845 (74.3)	17.9
EMPAREG OUTCOME 2019 [22, 23]	7020*	Empagliflozin 10 mg or 25 mg	357 (5.0)*	63.1*	3336 (71.2)*	7020 (100)*	74.0*	N/A	37.2
DECLARE TIMI 58 2018 [24]	3584	Dapagliflozin 10 mg	94 (2.6)	62	2739 ( 76.4)	3584 (100)	88.0	N/A	50.4
DACAMI 2023 [25]	100	Dapagliflozin 10 mg	N/A	56	83 (83)	0 (0)	84.0	100 (100)	2.8
DELIVER + DAPA-HF 2024 [26, 27]	3731	Dapagliflozin 10 mg	100 (2.7)	68.7	2825 (75.7)	1835 (49.2)	62.9	N/A	27.6
CANVAS 2021 [28, 29]	10,142*	Canagliflozin 100 mg or 300 mg	336 (3.3)*	63.2*	6509 ( 64.2)*	10,142 (100)*	76.5*	104 (24.7)*	43.3

Binary data is displayed as a number (%). ^†^Mean or Median; *Data reported from entire study population, not only myocardial infarction patients.SGLT2: sodium‒glucose cotransporter 2; DM II: type II diabetes; eGFR: estimated glomerular filtration rate; n: number; STEMI: ST-elevation myocardial infarction; y: year

### Pooled analysis of all studies

**Fig. 2:**
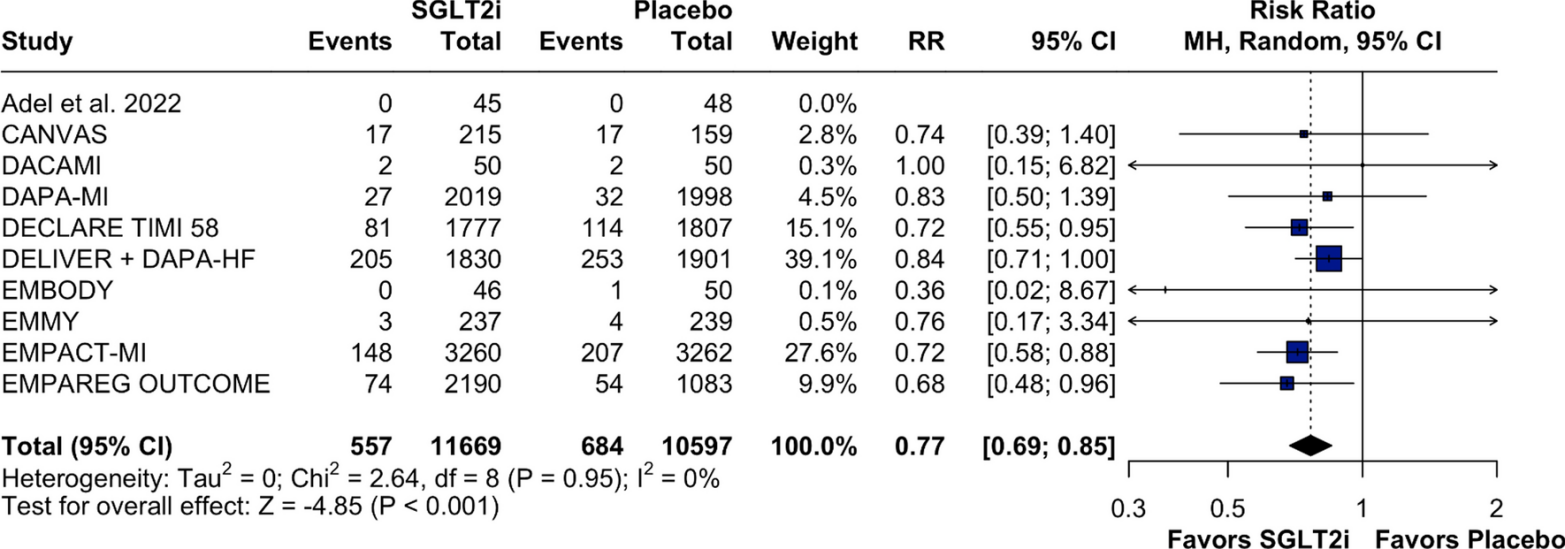
Forest plot for heart failure hospitalization. Legend: patients with MI events treated with SGLT2i had a lower risk of having HF hospitalization than did those treated with placebo. Abbreviations: CI confidence interval; HF: heart failure; MH: Mantel–Haenszel; MI: myocardial infarction; RR: risk ratio; SGLT2i: sodium–glucose-transporter-2 inhibitors

**Fig. 3:**
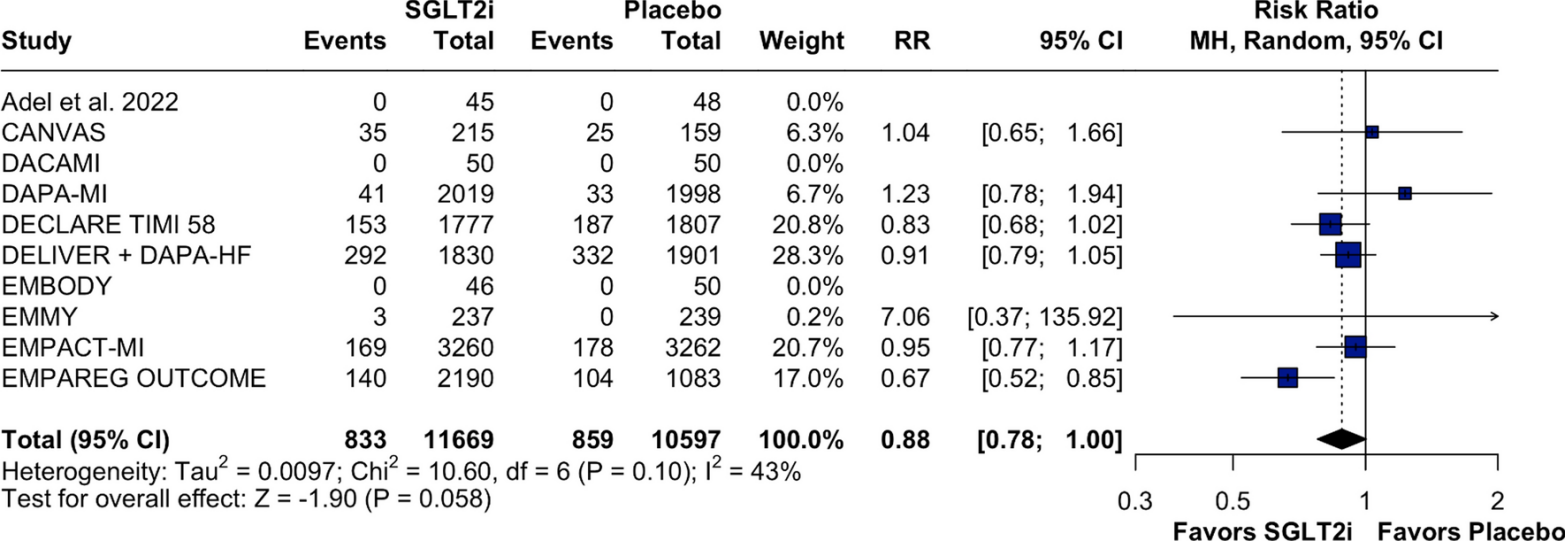
Forest plot for all-cause mortality. Legend: patients with MI events using SGLT2i had no significant change in all-cause mortality endpoint compared to placebo. Abbreviations: CI: confidence interval; MH: Mantel–Haenszel; MI: myocardial infarction; RR: risk ratio; SGLT2i: sodium–glucose-transporter-2 inhibitors

Patients treated with SGLT2 inhibitors experienced lower rates of hospitalizations for HF compared to those in the placebo group (RR: 0.77; 95% CI 0.69, 0.85; *p* < 0.001; I^2^:0%; Fig. 2), a result that was consistent in the time to first event analysis (HR: 0.75; 95% CI 0.67, 0.85; *p* < 0.01; *I*^2^:0%; Supplementary Fig. 1). For all-cause mortality, there appeared to be a trend towards reduction with SGLT2 inhibitors, although the difference was not statistically significant and there was moderate statistical heterogeneity (RR: 0.88; 95% CI 0.78, 1.00; *p* = 0.058; I^2^: 43%; Fig. 3). This result persisted when the analysis was done as a time to first event (HR: 0.87; 95% CI 0.75, 1.01; *p* = 0.07; *I*^2^: 53%; Supplementary Fig. 2).

**Fig. 4:**
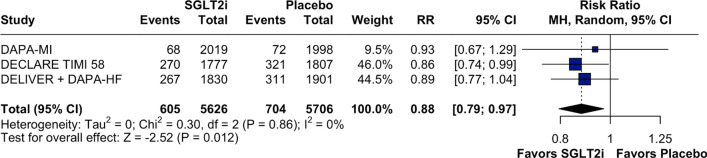
Forest plot for major cardiovascular events. Legend: patients with MI events treated with SGLT2i had a significant decrease in the MACE endpoint compared with those treated with placebo. Abbreviations: CI: confidence interval; MH: Mantel–Haenszel; MI: myocardial infarction; RR: risk ratio; SGLT2i: sodium–glucose-transporter-2 inhibitors

For MACE outcome, we observed significant benefits in favor of the SGLT2 inhibitor group in terms of the HR (Supplementary Fig. 3; HR 0.87; 95% CI 0.79, 0.97; *p* = 0.01; I^2^:0%) and RR (RR: 0.88; 95% CI 0.79, 0.97; *p* = 0.012; *I*^2^:0%; Fig. 4). However, there was no statistically significant difference between the groups in terms of the risk of recurrent MI (RR: 1.04; 95% CI 0.80, 1.36; *p* = 0.75; I^2^:68%; Supplementary Fig. 4A); (HR: 1.00; 95% CI 0.73, 1.37; *p* = 0.98; *I*^2^:73%; Supplementary Fig. 4B), CV death (RR: 0.91; 95% CI 0.75, 1.10; *p* = 0.32; I^2^:44%; Supplementary Fig. 5A); (HR: 0.87; 95% CI 0.71, 1.07; *p* = 0.19; *I*^2^:59%; Supplementary Fig. 5B), and stroke risk (RR: 0.88; 95% CI 0.65, 1.17; *p* = 0.37; I^2^:0%; Supplementary Fig. 6A); (HR: 0.87; 95% CI 0.64, 1.18; *p* = 0.37; *I*^2^:0%; Supplementary Fig. 6B).

### Prespecified subgroup analysis

**Fig. 5:**
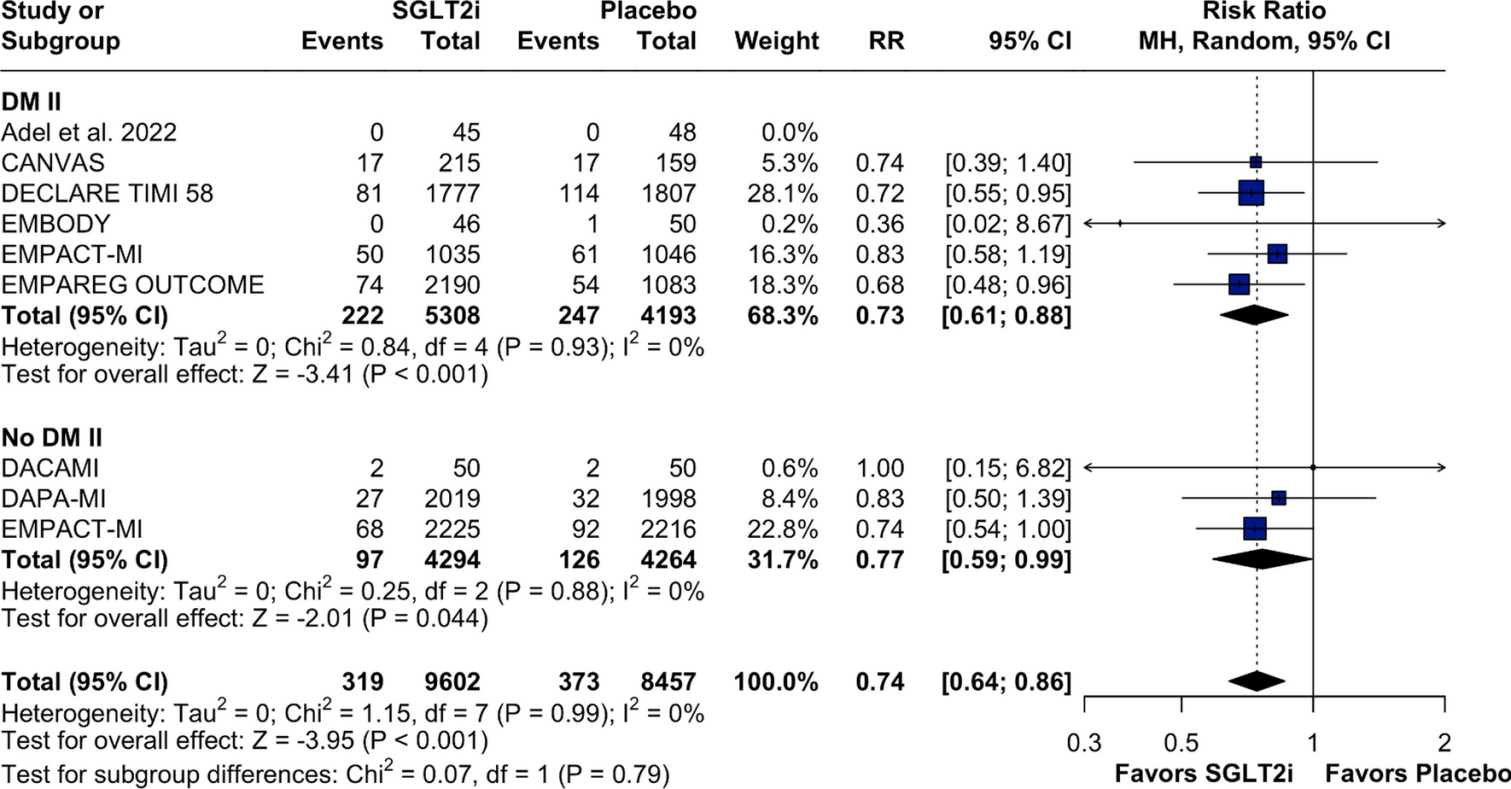
Forest plot for subgroup analysis in patients with DM II versus non-DM II. Legend: patients in the T2DM subgroup and those in the non-T2DM subgroup had no difference in the primary endpoint. Abbreviations: CI: confidence interval; DM II: diabetes mellitus type II; MH: Mantel–Haenszel; MI: myocardial infarction; RR: risk ratio; SGLT2i: sodium–glucose-transporter-2 inhibitors

**Fig. 6:**
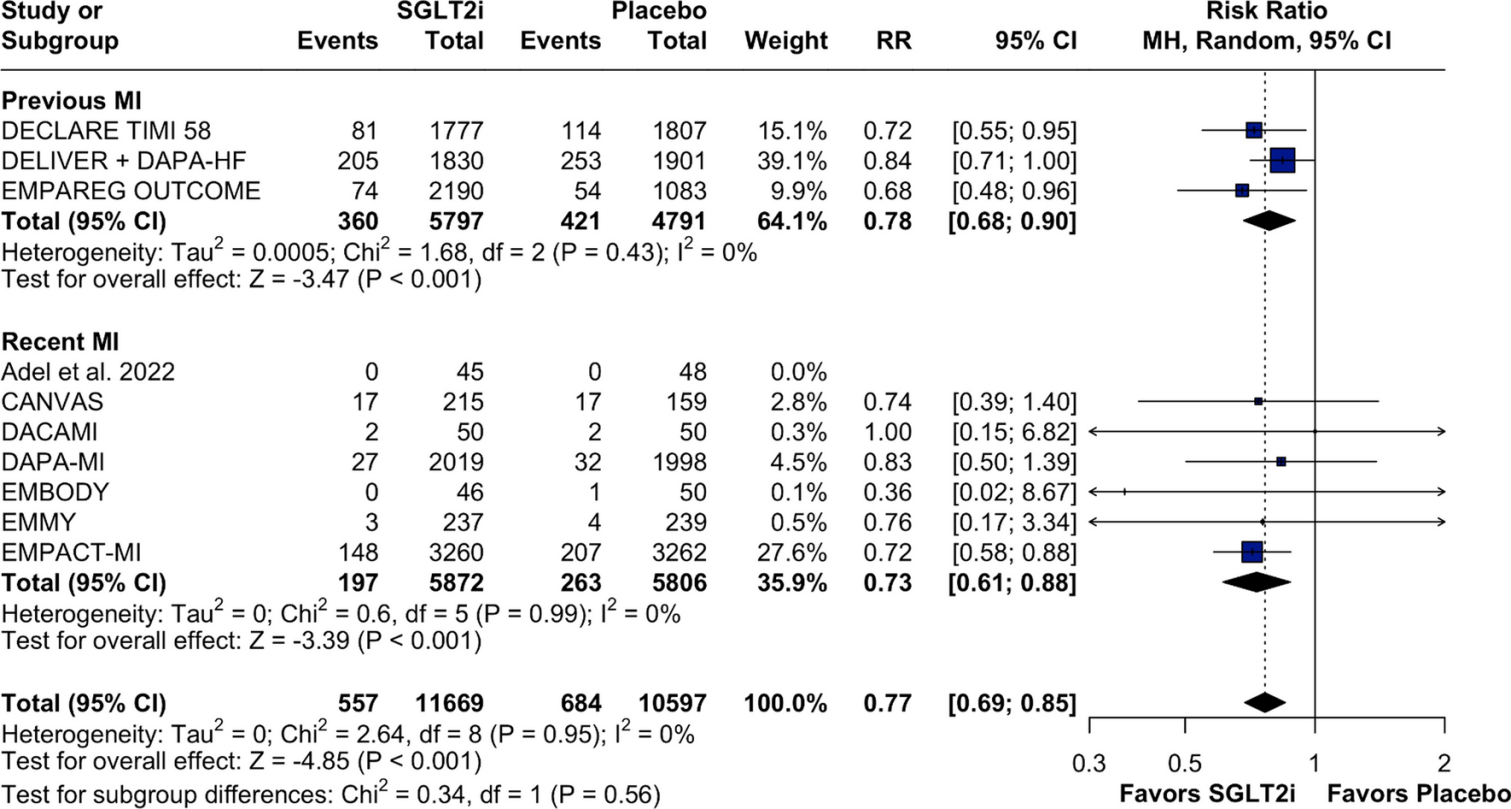
Forest plot for subgroup analysis in patients with recent MI versus previous MI. Legend: Patients with recent MI and previous MI subgroups showed no difference in the primary endpoint. Abbreviations: CI: confidence interval; MH: Mantel–Haenszel; MI: myocardial infarction; rr: risk ratio; SGLT2i: sodium–glucose-transporter-2 inhibitor

The subgroup analysis demonstrated that patients, both with and without T2DM, benefited from the use of SGLT2 inhibitors, with a significantly lower risk of HF hospitalizations. No significant heterogeneity in the effects of SGLT2 inhibitors was observed between these subgroups (Fig. 5; p for interaction = 0.79). Both patients with previous MI, defined as two or more months since the event, and those with recent MI, defined as less than two months since the event, appeared to benefit from SGLT2 inhibitors, with no evidence of heterogeneity in the treatment effect between subgroups, as shown in Fig. 6; p for interaction = 0.56. Additionally, empagliflozin and dapagliflozin demonstrated similar efficacy in reducing HF hospitalizations compared with placebo (Supplementary Fig. 7; p for interaction = 0.22).

### Sensitivity analysis and trial sequential analysis

We performed a sensitivity analysis using the leave‒one-out method for the outcomes of HF hospitalizations, all-cause mortality, CV death, and MI recurrence. Our primary outcome showed similar results after each trial was sequentially omitted (Supplementary Fig. 8A). On the other hand, after omitting the EMPAREG-OUTCOME trial, our sensitivity analysis revealed a nonsignificant reduction in all-cause mortality (RR: 0.92; 95% CI 0.84 to 1.02; I2 = 0%; Supplementary Fig. 8B) and CV death (RR: 0.95; 95% CI 0.84 to 1.07; I2:0%; Supplementary Fig. 8C), favoring the SGLT2 inhibitor group. Finally, for the MI recurrence endpoint, after the DECLARE-TIMI 58 trial was omitted, there was a non significant reduction in the risk (RR: 1.20; 95% CI 0.98, 1.47; I2:0%; Supplementary Fig. 8D) of SGLT-2 inhibitor therapy compared with placebo. In our post hoc sensitivity analysis for the primary endpoint in patients with an LVEF < 50% following acute MI, we found no significant difference between SGLT2 inhibitors and placebo (RR: 0.80; 95% CI 0.63, 1.04; I^2^ = 0%; Supplementary Fig. 9A). Similarly, for the HF hospitalization outcome in patients with STEMI, SGLT2 inhibitors had no significant effect compared with placebo (RR: 0.85; 95% CI 0.64, 1.12; I^2^ = 0%; Supplementary Fig. 9B). Furthermore, our sensitivity analysis after excluding prespecified analyses and post hoc studies was consistent with the overall findings and demonstrated a reduction in HF hospitalization rates (RR: 0.79; 95% CI 0.69, 0.90; I^2^ = 0%; Supplementary Fig. 9C). A TSA was conducted to ensure robust conclusions regarding the primary outcome. The TSA revealed a Z-curve that reached the required information size (RIS) and crossed the significance threshold, indicating a beneficial effect. Moreover, the TSA for subgroups also showed positive results (Supplementary Fig. 10A). A Z curve that exceeded a threshold indicated a benefit in patients with a history of T2DM and MI and those without a history of MI (Supplementary Fig. 10B to D). This suggests consistent benefits across these subgroups. However, the TSA for the subgroup of patients without a history of T2DM did not reach conclusive results (Supplementary Fig. 10E), and the Z-curve fell short of the RIS for 17,679 patients. TSA revealed a beneficial effect on MACE, but the analysis did not reach the RIS of 12,683 patients (Supplementary Fig. 10F).

### Quality assessment and publication bias

The RoB-2 tool was used for quality assessment. Adel et al.'s study was considered at moderate risk of bias [18], whereas the others remained at low risk, as described in the Supplementary Fig. 11. In the funnel plot analysis, studies presented a symmetrical distribution according to weight and converged toward the pooled effect as the weight increased, as described in the Supplementary Fig. 12. Egger’s test also revealed no evidence of publication bias (*p* = 0.82; Supplementary Fig. 12).

## Discussion

In this systematic review and meta-analysis of 10 RCTs enrolling 22,266 participants, we compared SGLT2 inhibitors versus placebo in patients with recent or previous MI. Our main results were as follows: (1) SGLT2 inhibitors reduced hospitalizations for HF; (2) this reduction in hospitalizations for HF with SGLT2 inhibitors was observed regardless of the timing of the MI; (3) HF hospitalization rates did not differ significantly between patients with and without T2DM when SGLT2 inhibitors were used; (4) SGLT2 inhibitors were associated with a lower incidence of MACE; and (5) there was no significant difference in the incidence of MI, CV death, or all-cause mortality between patients treated with SGLT2 inhibitors versus placebo.

Despite minimal SGLT2 expression in the heart, SGLT2 inhibitors significantly improve cardiac function by enhancing sodium handling and contractility, shifting myocardial energy use toward more efficient substrates, and reducing oxidative stress [30]. Recent RCTs have demonstrated that SGLT2 inhibitors provide CV benefits in various populations, being recognized not only for their glucose-lowering effects but also for their pleiotropic benefits, which include anti-inflammatory and plaque-stabilizing properties. These effects are particularly relevant for patients with complex CV conditions, such as multi-vessel coronary disease [31], a history of MI [32], acute MI patients undergoing PCI [33], and HF [34]. Therefore, SGLT2 inhibitors have acquired more space in the cardiology field, having been suggested as a class I recommendation for HF regardless of the ejection fraction by the European Society of Cardiology guidelines (ESC) [35]. SGLT2 inhibitors may also benefit patients without HF who have experienced MI [26] as they seem to reduce in-hospital arrhythmias [36] and contrast-induced acute kidney injury (CI-AKI) [37] in the post-MI setting. These promising effects have driven further exploration of the role of SGLT2 inhibitors in patients with MI and established coronary artery disease.

The main outcome of our meta-analysis underscores the lower rates of HF hospitalizations with SGLT2 inhibitors in patients following MI. A previous study [38] by Jenca et al. demonstrated that, within one year after an MI, 20 to 30% of patients are diagnosed with late-onset HF. The EMMY trial revealed no significant difference in HF hospitalizations with SGLT2 use in acute MI patients (RR 0.76; 95% CI 0.17, 3.34). However, as noted in the study's limitations, the sample size in this trial was insufficient to provide adequate power for hard clinical endpoints. Our meta-analysis aligns with the results of major RCTs: the EMPACT-MI trial [20] by Butler et al. included 6522 post-MI patients at high CV risk, showing that those treated with empagliflozin experienced fewer HF hospitalizations (2.4 events per 100 patient-years) than those receiving a placebo did (3.6 events per 100 patient-years). Notably, HF hospitalization was a secondary outcome, and the primary outcome, a composite of all-cause mortality or HF hospitalization, was not reduced by empagliflozin. Additionally, SGLT2 inhibitors have been shown to attenuate cardiac remodeling after MI by reducing cardiac fibrosis [39, 40]. Therefore, regarding HF hospitalizations, these results demonstrated that these drugs could significantly impact a patient's prognosis after MI. Moreover, MACE was also lower in the SGLT2 inhibitor group (*p* = 0.012). The DECLARE TIMI 58 trial [24] compared dapagliflozin with placebo in patients with a history of MI and T2DM, and their results were similar to our analysis (HR 0.84 95% CI 0.72, 0.99). These findings underscore the importance of SGLT2 inhibitor therapy for patients with MI, as it may reduce CV outcomes.

Drugs widely known to reduce mortality in patients with HF, such as beta-blockers [41], angiotensin-converting enzyme (ACE) inhibitors and mineralocorticoid antagonists [42], have demonstrated benefits when started in the acute phase of MI, whereas others, such as sacubitril-valsartan, have not [43], raising concerns about the optimal timing for initiating SGLT2 inhibitors after MI at high risk of developing HF. Patients with a history of previous MI may exhibit distinct baseline characteristics and risk profiles compared with those experiencing recent MI, which can lead to variability in treatment responses. However, including patients with both recent and previous MI can increase the generalizability of the findings, as they mirror real-world clinical scenarios where patients often present with diverse histories of CV events. Although patients who experienced recent MI may be at greater risk for developing CV death and HF events, our analysis demonstrated that SGLT2 inhibitors were also effective in lowering HF hospitalization rates in patients with previous MI (*p* = 0.56). This approach highlights the potential importance of early intervention, suggesting that incorporating SGLT2 inhibitors into the treatment plans of patients with a history of prior MI may contribute to improved long-term CV health. Our TSA analysis indicated that our meta-analysis met the RIS, supporting the robustness of our findings.

A limitation of previous studies is the lack of data regarding the effect of SGLT2 inhibitors in patients without T2DM. Only 3 out of 10 studies in our meta-analysis included patients without T2DM. The DAPA-AMI trial, which included 4017 patients without T2DM [17] and high CV risk, reported similar hospitalization for HF rates in dapagliflozin and placebo groups (1.3% vs 1.6%; RR 0.83 95% CI 0.5, 1.39). These findings were not consistent with those of a subgroup analysis of patients with (HR 0.91 95% CI 0.63, 1.32) and without diabetes (HR 0.68 95% CI 0.50, 0.93) in the EMPACT-MI trial [21]. Therefore, we performed a subgroup analysis of HF hospitalization by comparing patients with and without T2DM. Our results suggested similar efficacy for both subgroups when treated with SGLT2 inhibitors. However, our TSA results suggested that, in patients without -T2DM, the conventional boundary for RIS was not met, requiring a larger sample size in this subgroup to confirm our findings. This suggests that additional data are needed to confirm the positive trend and draw a definitive conclusion for patients without T2DM.

Additionally, despite the high heterogeneity, our meta-analysis indicated a comparable reduction in all-cause and CV mortality between the SGLT2 inhibitor and placebo groups across trials. Our leave-one-out sensitivity analysis showed that excluding the EMPA-REG OUTCOME trial [22, 23] reduced heterogeneity to 0% for both mortality endpoints. This could be attributed to the trial design, which included patients across a broad CV risk spectrum. Since most post-MI patients were classified as high risk, this may have reduced the observable impact of SGLT2 inhibitors on mortality outcomes. Therefore, further large-scale studies are needed to clarify the effects of SGLT2 inhibitors on CV events and all-cause mortality.

In our meta-analysis, risk of recurrent MI was not different between SGLT2 inhibitors and placebo. The contrasting results observed for this outcome can be partially explained by the post hoc analysis of the CANVAS and CREDENCE programs [29] The CANVAS program tested canagliflozin (100; 300 mg) in patients with previous MI and T2DM and high CV risk. Patients treated with canagliflozin had a substantial increase in MI incidence, and the rate of STEMI was also higher among individuals randomized to canagliflozin than among those randomized to placebo (32% vs 15%; *p* < 0.001), whereas non-ST-elevation myocardial infarction (NSTEMI) was lower (63% vs. 78%, *p* < 0.001). The exact mechanism by which canagliflozin demonstrated these results remains unknown but may be related to increase in hematocrit and in blood viscosity caused by SGLT2 inhibitors. Nevertheless, the DECLARE TIMI 58 [24] trial highlighted the reduction in overall rates of MI in patients with previous MI treated with SGLT2 inhibitors. A further subgroup analysis revealed significantly lower rates of type 2 MI (HR 0.64 95% CI 0.42, 0.97), although no corresponding reduction was observed for type I MI. This result demonstrates an important advance in therapies for reducing type 2 MI, considering that few medications have presented positive results for this population.

Our meta-analysis has several significant strengths compared with previous meta-analyses, [8, 9] that explored the effects of SGLT2 inhibitors in acute MI. We addressed several gaps in the literature with our meta-analysis. First, we included five new studies with additional 11,062 patients, enhancing the robustness of our data. Second, our TSA confirmed that the RIS was met, providing sufficient evidence to support the benefits of SGLT2 inhibitors. Third, our subgroup analysis comparing patients with recent versus previous MI revealed that SGLT2 inhibitors showed similar benefits in reducing HF hospitalizations in both groups. This finding helps to resolve a key question in the literature regarding the timing of SGLT2 inhibitor initiation post-MI. Fourth, the MACE outcome rates were lower with SGLT2 inhibitors. Despite the limited number of trials addressing this outcome, these results offer valuable insights into the role of SGLT2 inhibitors in CV care. Finally, we included a time to first event analysis, which was consistent with our major finding.

Our meta-analysis has limitations that warrant consideration. The data from the EMPA-REG OUTCOME and CANVAS trials presented in Table 1 are derived from the original studies [22, 28] rather than the secondary analyses [23, 29] used in the statistical report, which may have led to variability in defining our target population by including patients with atherosclerosis and a history of myocardial infarction (MI), rather than exclusively those with MI. Furthermore, the follow-up duration varied significantly across studies, ranging from 2.8 to 50.4 months, highlighting the need for further RCTs with longer follow-up periods. The incidence of HF hospitalizations may also have been underestimated in two trials [17, 21] due to disruptions caused by the COVID-19 pandemic. Although our TSA of HF hospitalizations demonstrated statistically significant benefits for patients with T2DM, it did not yield significant results for patients without T2DM, leaving an important gap in our findings. Another limitation was the variation of drug dosages investigated across the included studies, as outlined in Table 1, which could introduce heterogeneity in the analysis. Additionally, while MACE definitions vary widely across the literature, all studies in our analysis that evaluated this outcome consistently used the same definition, ensuring a more homogeneous assessment. Moreover, the inclusion of two subgroup analysis [23, 24] from two trials not originally dedicated to the post MI population is another limitation, although we did a sensitivity analysis excluding both and the results remained consistent with the overall analysis. Finally, the inclusion of a post hoc analysis from the CANVAS and CREDENCE programs [29] was restricted to data from CANVAS patients, as relevant outcomes from the CREDENCE program were unavailable.

## Conclusion

This systematic review and meta-analysis explored the potential efficacy of SGLT2 inhibitors in patients with MI. This therapy was associated with benefits regarding hospitalization for HF and MACE in patients with both recent and previous MI. These findings suggest that SGLT2 inhibitors might be considered not only in patients with acute MI but also in those with atherosclerosis and a history of previous MI.

## Supplementary Information

Additional file 1.

## Data Availability

No datasets were generated or analysed during the current study.

## Abbreviations

ACE: Angiotensin-converting enzyme
CI: Confidence interval
CI-AKI: Contrast-induced acute kidney injury
CV: Cardiovascular
ESC: European Society of Cardiology
HF: Heart failure
HR: Hazard ratio
LVEF: Left ventricular ejection fraction
MACE: Major adverse cardiovascular events
MI: Myocardial infarction
NSTEMI: Non-ST-elevation myocardial infarction
PRISMA: Preferred Reporting Items for Systematic Reviews and Meta-Analysis
PCI: Percutaneous Coronary Intervention
PROSPERO: Prospective Register of Systematic Reviews
RCT: Randomized controlled trial
RIS: Required information size
RoB-2: Revised Cochrane risk of bias tool for randomized trials
RR: Risk ratio
SGLT2i: Sodium–glucose cotransporter-2 inhibitor
SGLT2: Sodium‒glucose cotransporter-2
STEMI: ST-elevation myocardial infarction
TSA: Trial sequential analysis
T2DM: Type 2 diabetes mellitus

## References

[1] McAloon CJ, Boylan LM, Hamborg T, Stallard N, Osman F, Lim PB, et al. The changing face of cardiovascular disease 2000–2012: an analysis of the world health organisation global health estimates data. Int J Cardiol. 2016;1(224):256–64. 10.1016/j.ijcard.2016.09.026 27664572

[2] Saito Y, Oyama K, Tsujita K, Yasuda S, Kobayashi Y. Treatment strategies of acute myocardial infarction: updates on revascularization, pharmacological therapy, and beyond. J Cardiol. 2023;81(2):168–78. 35882613 10.1016/j.jjcc.2022.07.003

[3] Wang Y, Leifheit EC, Krumholz HM. Trends in 10-year outcomes among medicare beneficiaries who survived an acute myocardial infarction. JAMA Cardiol. 2022;7(6):613–22. 35507330 10.1001/jamacardio.2022.0662 PMC9069341

[4] Heidenreich PA, Bozkurt B, Aguilar D, Allen LA, Byun JJ, Colvin MM, et al. AHA/ACC/HFSA guideline for the management of heart failure: a report of the American College of Cardiology/American Heart Association Joint Committee on Clinical Practice Guidelines. J Am Coll Cardiol. 2022;79(17):e263-421. 35379503 10.1016/j.jacc.2021.12.012

[5] Heerspink HJL, Stefánsson BV, Correa-Rotter R, Chertow GM, Greene T, Hou FF, et al. Dapagliflozin in patients with chronic kidney disease. N Engl J Med. 2020;383(15):1436–46. 32970396 10.1056/NEJMoa2024816

[6] The EMPA-KIDNEY Collaborative Group, Herrington WG, Staplin N, Wanner C, Green JB, Hauske SJ, et al. Empagliflozin in Patients with Chronic Kidney Disease. N Engl J Med. 2023;388(2):117–27. 10.1056/NEJMoa2204233 PMC7614055 36331190

[7] Zelniker TA, Wiviott SD, Raz I, Im K, Goodrich EL, Bonaca MP, et al. SGLT2 inhibitors for primary and secondary prevention of cardiovascular and renal outcomes in type 2 diabetes: a systematic review and meta-analysis of cardiovascular outcome trials. Lancet. 2019;393(10166):31–9. 30424892 10.1016/S0140-6736(18)32590-X

[8] Idowu A, Adebolu O, Wattanachayakul P, Obomanu E, Shah S, Lo KB, et al. Cardiovascular outcomes of sodium–glucose co-transporter 2 inhibitors use after myocardial infarction: a systematic review and meta-analysis of randomized controlled trials. Curr Probl Cardiol. 2024;49(8):102648. 38759767 10.1016/j.cpcardiol.2024.102648

[9] Nall S, Rawat A, Gill FS, Saleem R, Saeed S, Ahmed S, et al. Assessing the effect of sodium–glucose cotransporter 2 inhibitor (SGLT2i) on outcomes in patients with acute myocardial infarction: a systematic review and meta-analysis. Cureus. 2024;16(6):e62978. 39050303 10.7759/cureus.62978 PMC11265972

[10] Page MJ, McKenzie JE, Bossuyt PM, Boutron I, Hoffmann TC, Mulrow CD, et al. The PRISMA 2020 statement: an updated guideline for reporting systematic reviews. BMJ. 2021;29(372): n71. 10.1136/bmj.n71 PMC8005924 33782057

[11] Higgins JPT, Thomas J, Chandler J, Cumpston M, Li T, Page MJ, Welch VA, eds. Cochrane handbook for systematic reviews of interventions. Version 6.4. Updated August 2023. Cochrane; 2023. www.training.cochrane.org/handbook. n.d.

[12] Kramer CM, Rogers WJ, Theobald TM, Power TP, Geskin G, Reichek N. Dissociation between changes in intramyocardial function and left ventricular volumes in the eight weeks after first anterior myocardial infarction. J Am Coll Cardiol. 1997;30(7):1625–32. 9385886 10.1016/s0735-1097(97)00406-3

[13] Sterne JAC, Savović J, Page MJ, Elbers RG, Blencowe NS, Boutron I, et al. RoB 2: a revised tool for assessing risk of bias in randomised trials. BMJ. 2019;28(366):l4898. 10.1136/bmj.l4898 31462531

[14] Sterne JAC, Sutton AJ, Ioannidis JPA, Terrin N, Jones DR, Lau J, et al. Recommendations for examining and interpreting funnel plot asymmetry in meta-analyses of randomised controlled trials. BMJ. 2011;22(343):d4002. 10.1136/bmj.d4002 21784880

[15] Balduzzi S, Rücker G, Schwarzer G. How to perform a meta-analysis with R: a practical tutorial. Evid Based Ment Health. 2019;22(4):153–60. 31563865 10.1136/ebmental-2019-300117 PMC10231495

[16] Trial Sequential Analysis (TSA) [Computer program]. Version 0.9.5.10 Beta. The Copenhagen Trial Unit, Centre for Clinical Intervention Research, The Capital Region, Copenhagen University Hospital—Rigshospitalet; 2021. n.d.

[17] James S, Erlinge D, Storey RF, McGuire DK, de Belder M, Eriksson N, et al. Dapagliflozin in myocardial infarction without diabetes or heart failure. NEJM Evid. 2024;3(2):EVIDoa3200286. 10.1056/EVIDoa2300286 38320489

[18] Adel SMH, Jorfi F, Mombeini H, Rashidi H, Fazeli S. Effect of a low dose of empagliflozin on short-term outcomes in type 2 diabetics with acute coronary syndrome after percutaneous coronary intervention. Saudi Med J. 2022;43(5):458–64. 35537720 10.15537/smj.2022.43.5.20220018 PMC9280595

[19] Shimizu W, Kubota Y, Hoshika Y, Mozawa K, Tara S, Tokita Y, et al. Effects of empagliflozin versus placebo on cardiac sympathetic activity in acute myocardial infarction patients with type 2 diabetes mellitus: the EMBODY trial. Cardiovasc Diabetol. 2020;19(1):148. 32977831 10.1186/s12933-020-01127-z PMC7519555

[20] von Lewinski D, Kolesnik E, Tripolt NJ, Pferschy PN, Benedikt M, Wallner M, et al. Empagliflozin in acute myocardial infarction: the EMMY trial. Eur Heart J. 2022;43(41):4421–32. 36036746 10.1093/eurheartj/ehac494 PMC9622301

[21] Butler J, Jones WS, Udell JA, Anker SD, Petrie MC, Harrington J, et al. Empagliflozin after acute myocardial infarction. N Engl J Med. 2024;390(16):1455–66. 38587237 10.1056/NEJMoa2314051

[22] Zinman B, Wanner C, Lachin JM, Fitchett D, Bluhmki E, Hantel S, et al. Empagliflozin, cardiovascular outcomes, and mortality in type 2 diabetes. N Engl J Med. 2015;373(22):2117–28. 26378978 10.1056/NEJMoa1504720

[23] Fitchett D, Inzucchi SE, Cannon CP, McGuire DK, Scirica BM, Johansen OE, et al. Empagliflozin reduced mortality and hospitalization for heart failure across the spectrum of cardiovascular risk in the EMPA-REG OUTCOME Trial. Circulation. 2019;139(11):1384–95. 30586757 10.1161/CIRCULATIONAHA.118.037778 PMC6416009

[24] Furtado RHM, Bonaca MP, Raz I, Zelniker TA, Mosenzon O, Cahn A, et al. Dapagliflozin and cardiovascular outcomes in patients with type 2 diabetes mellitus and previous myocardial infarction. Circulation. 2019;139(22):2516–27. 30882239 10.1161/CIRCULATIONAHA.119.039996

[25] Dayem KA, Younis O, Zarif B, Attia S, AbdelSalam A. Impact of dapagliflozin on cardiac function following anterior myocardial infarction in non-diabetic patients—DACAMI (a randomized controlled clinical trial). Int J Cardiol. 2023;15(379):9–14. 10.1016/j.ijcard.2023.03.002 36889650

[26] Solomon SD, Vaduganathan M, Claggett BL, de Boer RA, DeMets D, Hernandez AF, et al. Baseline characteristics of patients with HF with mildly reduced and preserved ejection fraction: DELIVER Trial. JACC Heart Fail. 2022;10(3):184–97. 35241246 10.1016/j.jchf.2021.11.006

[27] Peikert A, Vaduganathan M, Claggett BL, Kulac IJ, Foà A, Desai AS, et al. Dapagliflozin in patients with heart failure and previous myocardial infarction: a participant-level pooled analysis of DAPA-HF and DELIVER. Eur J Heart Fail. 2024;26(4):912–24. 38487939 10.1002/ejhf.3184

[28] Neal B, Perkovic V, Mahaffey KW, de Zeeuw D, Fulcher G, Erondu N, et al. Canagliflozin and cardiovascular and renal events in type 2 diabetes. N Engl J Med. 2017;377(7):644–57. 28605608 10.1056/NEJMoa1611925

[29] Yu J, Li J, Leaver PJ, Arnott C, Huffman MD, Udell JA, et al. Effects of canagliflozin on myocardial infarction: a post hoc analysis of the CANVAS programme and CREDENCE trial. Cardiovasc Res. 2022;118(4):1103–14. 33826709 10.1093/cvr/cvab128

[30] Marfella R, Scisciola L, D’Onofrio N, Maiello C, Trotta MC, Sardu C, et al. Sodium–glucose cotransporter-2 (SGLT2) expression in diabetic and non-diabetic failing human cardiomyocytes. Pharmacol Res. 2022;184:106448. 36096423 10.1016/j.phrs.2022.106448

[31] Sardu C, Trotta MC, Sasso FC, Sacra C, Carpinella G, Mauro C, et al. SGLT2-inhibitors effects on the coronary fibrous cap thickness and MACEs in diabetic patients with inducible myocardial ischemia and multi vessels non-obstructive coronary artery stenosis. Cardiovasc Diabetol. 2023;22(1):80. 37005586 10.1186/s12933-023-01814-7 PMC10067292

[32] Sardu C, Massetti M, Testa N, Martino LD, Castellano G, Turriziani F, et al. Effects of Sodium–glucose transporter 2 inhibitors (SGLT2-I) in patients with ischemic heart disease (IHD) treated by coronary artery bypass grafting via MiECC: inflammatory burden, and clinical outcomes at 5 years of follow-up. Front Pharmacol. 2021;12:777083. 34867407 10.3389/fphar.2021.777083 PMC8634684

[33] Paolisso P, Bergamaschi L, Gragnano F, Gallinoro E, Cesaro A, Sardu C, et al. Outcomes in diabetic patients treated with SGLT2-inhibitors with acute myocardial infarction undergoing PCI the SGLT2-I AMI PROTECT Registry. Pharmacol Res. 2023;187:106597. 36470546 10.1016/j.phrs.2022.106597 PMC9946774

[34] Mentz RJ, Brunton SA, Rangaswami J. Sodium–glucose cotransporter-2 inhibition for heart failure with preserved ejection fraction and chronic kidney disease with or without type 2 diabetes mellitus: a narrative review. Cardiovasc Diabetol. 2023;22(1):316. 37974185 10.1186/s12933-023-02023-y PMC10655322

[35] McDonagh TA, Metra M, Adamo M, Gardner RS, Baumbach A, Böhm M, et al. Focused update of the 2021 ESC guidelines for the diagnosis and treatment of acute and chronic heart failure. Eur Heart J. 2023;44(37):3627–39. 37622666 10.1093/eurheartj/ehad195

[36] Cesaro A, Gragnano F, Paolisso P, Bergamaschi L, Gallinoro E, Sardu C, et al. In-hospital arrhythmic burden reduction in diabetic patients with acute myocardial infarction treated with SGLT2-inhibitors: Insights from the SGLT2-I AMI PROTECT study. Front Cardiovasc Med. 2022;9:1012220. 36237914 10.3389/fcvm.2022.1012220 PMC9551177

[37] Paolisso P, Bergamaschi L, Cesaro A, Gallinoro E, Gragnano F, Sardu C, et al. Impact of SGLT2-inhibitors on contrast-induced acute kidney injury in diabetic patients with acute myocardial infarction with and without chronic kidney disease: insight from SGLT2-I AMI PROTECT registry. Diabetes Res Clin Pract. 2023;202:110766. 37276980 10.1016/j.diabres.2023.110766

[38] Jenča D, Melenovský V, Stehlik J, Staněk V, Kettner J, Kautzner J, et al. Heart failure after myocardial infarction: incidence and predictors. ESC Heart Fail. 2021;8(1):222–37. 33319509 10.1002/ehf2.13144 PMC7835562

[39] Verma S, McMurray JJV. SGLT2 inhibitors and mechanisms of cardiovascular benefit: a state-of-the-art review. Diabetologia. 2018;61(10):2108–17. 30132036 10.1007/s00125-018-4670-7

[40] Frantz S, Hundertmark MJ, Schulz-Menger J, Bengel FM, Bauersachs J. Left ventricular remodelling post-myocardial infarction: pathophysiology, imaging, and novel therapies. Eur Heart J. 2022;43(27):2549–61. 35511857 10.1093/eurheartj/ehac223 PMC9336586

[41] Dargie HJ. Effect of carvedilol on outcome after myocardial infarction in patients with left-ventricular dysfunction: the CAPRICORN randomised trial. Lancet. 2001;357(9266):1385–90. 11356434 10.1016/s0140-6736(00)04560-8

[42] Pitt B, Remme W, Zannad F, Neaton J, Martinez F, Roniker B, et al. Eplerenone, a selective aldosterone blocker, in patients with left ventricular dysfunction after myocardial infarction. N Engl J Med. 2003;348(14):1309–21. 12668699 10.1056/NEJMoa030207

[43] Pfeffer MA, Claggett B, Lewis EF, Granger CB, Køber L, Maggioni AP, et al. Angiotensin receptor-neprilysin inhibition in acute myocardial infarction. N Engl J Med. 2021;385(20):1845–55. 34758252 10.1056/NEJMoa2104508

