# Supplementary material for: Impact of sodium‒glucose cotransporter‐2 inhibitors in patients with recent versus previous myocardial infarction: a systematic review and meta-analysis
Source: Cardiovasc Diabetol. 2025 Feb 13;24:73. doi: 10.1186/s12933-024-02540-4 (PMC11827181; doi:10.1186/s12933-024-02540-4)
Supplement: Supplementary file 1 — Additional file 1. [file 12933_2024_2540_MOESM1_ESM.docx]

**SUPPLEMENTARY APPENDIX**

**Table of Contents**

[**Supplementary Methods 1. PRISMA 2020 Main Checklist 3**](#_2s8eyo1)

[**Supplementary Methods 2. PRISMA Abstract Checklist**](#_17dp8vu) **7**

[**Supplementary Methods 3. Details of the Search Strategy**](#_3rdcrjn) **9**

[**Supplementary Methods 4. Definitions of Outcomes**](#_26in1rg) **11**

[**Supplementary Table 1. Eligibility Criteria Used by Study**](#_lnxbz9) **12**

[**Supplementary Table 2. Additional Studies Characteristics 2**](#_ls1y3gbmdpzp)**8**

[**Supplementary Figure 1. Forest Plot for Time to Event Heart Failure Hospitalization 2**](https://docs.google.com/document/d/1SO5gJi59LIhPT4Ak83Y_7z7ahYDkpyC1/edit#heading=h.35nkun2)**9**

[**Supplementary Figure 2. Forest Plot for Time to All-Cause Mortality**](#_1ksv4uv) **30** [**Supplementary Figure 3. Forest Plot for Time to Event Major Adverse Cardiovascular Events 31**](#_44sinio)

[**Supplementary Figure 4. Forest Plot for Myocardial Infarction Endpoint**](#_2jxsxqh) **32**

[**Figure 4A. Forest Plot for Risk of MI Recurrence**](#_5wmvbr769145) **32**

[**Figure 4B. Forest Plot for Time to Event of MI Recurrence**](#_qu7fruwjzfdy) **32**

[**Supplementary Figure 5. Forest Plot for Cardiovascular Death Endpoint**](#_z337ya) **33**

[**Figure 5A. Forest Plot for risk of CV Death**](#_ze6zs0ohz62h) **33**

[**Figure 5B. Forest Plot for Time to Event of CV Death**](#_wrkpk09fnc0q) **33**

[**Supplementary Figure 6. Forest Plot for Stroke Endpoint**](#_3j2qqm3) **34**

[**Figure 6A. Forest Plot for risk of Stroke**](#_8txmyksnb9jk) **34**

[**Figure 6B. Forest Plot for Time to Event of Stroke**](#_q19oogg765hc) **34**

[**Supplementary Figure 7. Forest Plot for Subgroup Analysis for Empagliflozin versus Dapagliflozin**](#_1y810tw) **35**

[**Supplementary Figure 8. Sensitivity Analyses**](#_4i7ojhp) **36**

[**Figure 8A. Leave one out analysis for HF Hospitalization 3**](#_krvdgaorjb6l)**6**

[**Figure 8B. Leave one out analysis for All-Cause Mortality 3**](#_33z8zi2hj4x3)**6**

[**Figure 8C. Leave one out analysis for CV Death 3**](#_6822b2cben0v)**7**

[**Figure 8D. Leave one out analysis for MI recurrence 3**](#_mvnktg1xlxpf)**7**

[**Supplementary Figure 9. Post Hoc Sensitivity Analyses for the Primary Endpoint 3**](#_4i7ojhp)**8**

[**Figure 9A. Forest Plot for patients with LVEF<50% in acute MI setting 3**](#_krvdgaorjb6l)**8**

[**Figure 9B. Forest Plot for patients with STEMI in acute MI setting**](#_33z8zi2hj4x3) **38**

**Figure 9C. Sensitivity Analysis after removing pre specified analysis or *post hoc* studies**  [**3**](#_33z8zi2hj4x3)**8**

[**Supplementary Figure 10. Trial Sequential Analysis 3**](#_fwg5bwf3jyv1)**9**

[**Figure 10A. Trial Sequential Analysis for the primary endpoint 3**](#_iljesiho0fbz)**9**

[**Figure 10B. Trial Sequential Analysis for the previous MI subgroup of the primary endpoint 3**](#_ul61wlffdceb)**9**

[**Figure 10C. Trial Sequential Analysis for the new onset MI subgroup of the primary endpoint**](#_9zvfedt11a5d) **40**

[**Figure 10D. Trial Sequential Analysis for the DM II subgroup of the primary endpoint**](#_8lyxjkbd1g7v) **41**

[**Figure 10E. Trial Sequential Analysis for the non-DM II subgroup of the primary endpoint**](#_e4bybweg99fo) **42**

[**Figure 10F. Trial Sequential Analysis for the MACE Endpoint**](#_dehrwpahej6v) **42**

[**Supplementary Figure 11. Risk of Bias 2 of All Included Studies**](#_g520hfqku2eg) **43**

[**Figure 11A. "Traffic light" plot of the domain-level judgments for each study**](#_998rmnqud6ip) **43**

[**Figure 11B. Summary of overall weighted bar plot of risk-of-bias judgments within each bias domain.**](#_1xo3ic2ra34x) **43**

[**Supplementary Figure 12.Funnel Plot and Egger’s Test for the Primary Efficacy Endpoint**](#_2xcytpi) **44**

**Supplemental Methods 1. PRISMA 2020 Main Checklist**

| **Topic** | **No.** | **Item** | **Location where item is reported** |
| --- | --- | --- | --- |
| **TITLE** |  |  |  |
| **Title** | 1 | Identify the report as a systematic review. | Pg.1;MS |
| **ABSTRACT** |  |  |  |
| **Abstract** | 2 | See the PRISMA 2020 for Abstracts checklist | Pg.2; MS |
| **INTRODUCTION** |  |  |  |
| **Rationale** | 3 | Describe the rationale for the review in the context of existing knowledge. | Pg.4; MS |
| **Objectives** | 4 | Provide an explicit statement of the objective(s) or question(s) the review addresses. | Pg.4; MS |
| **METHODS** |  |  |  |
| **Eligibility criteria** | 5 | Specify the inclusion and exclusion criteria for the review and how studies were grouped for the syntheses. | Pg 5; MS |
| **Information sources** | 6 | Specify all databases, registers, websites, organizations, reference lists and other sources searched or consulted to identify studies. Specify the date when each source was last searched or consulted. | Pg. 5; MS |
| **Search strategy** | 7 | Present the full search strategies for all databases, registers, and websites, including any filters and limits used. | Pg. 5; MS and and Supp.Methods 3 |
| **Selection process** | 8 | Specify the methods used to decide whether a study met the inclusion criteria of the review, including how many reviewers screened each record and each report retrieved, whether they worked independently, and if applicable, details of automation tools used in the process. | Pg. 5; MS |
| **Data collection process** | 9 | Specify the methods used to collect data from reports, including how many reviewers collected data from each report, whether they worked independently, any processes for obtaining or confirming data from study investigators, and if applicable, details of automation tools used in the process. | Pg. 5; MS and and Supp.Methods 4 |
| **Data items** | 10a | List and define all outcomes for which data were sought. Specify whether all results that were compatible with each outcome domain in each study were sought (e.g., for all measures, time points, analyses), and if not, the methods used to decide which results to collect. | Pg. 5; MS |
|  | 10b | List and define all other variables for which data were sought (e.g., participant and intervention characteristics, funding sources). Describe any assumptions made about any missing or unclear information. | Pg. 5; MS |
| **Study risk of bias assessment** | 11 | Specify the methods used to assess risk of bias in the included studies, including details of the tool(s) used, how many reviewers assessed each study and whether they worked independently, and if applicable, details of automation tools used in the process. | Pg. 6; MS |
| **Effect measures** | 12 | Specify for each outcome the effect measure(s) (e.g., risk ratio, mean difference) used in the synthesis or presentation of results. | Pg. 6; MS |
| **Synthesis methods** | 13a | Describe the processes used to decide which studies were eligible for each synthesis (e.g., tabulating the study intervention characteristics and comparing against the planned groups for each synthesis (item 5)). | Table 1 |
|  | 13b | Describe any methods required to prepare the data for presentation or synthesis, such as handling of missing summary statistics, or data conversions. | Pg. 6; MS |
|  | 13c | Describe any methods used to tabulate or visually display results of individual studies and syntheses. | Pg. 6; MS |
|  | 13d | Describe any methods used to synthesize results and provide a rationale for the choice(s). If meta-analysis was performed, describe the model(s), method(s) to identify the presence and extent of statistical heterogeneity, and software package(s) used. | Pg. 6; MS |
|  | 13e | Describe any methods used to explore possible causes of heterogeneity among study results (e.g., subgroup analysis, meta-regression). | Pg. 6-7;MS |
|  | 13f | Describe any sensitivity analyses conducted to assess robustness of the synthesized results. | Pg. 6-7;MS |
| **Reporting bias assessment** | 14 | Describe any methods used to assess risk of bias due to missing results in a synthesis (arising from reporting biases). | Pg. 6; MS |
| **Certainty assessment** | 15 | Describe any methods used to assess certainty (or confidence) in the body of evidence for an outcome. | NA |
| **RESULTS** |  |  |  |
| **Study selection** | 16a | Describe the results of the search and selection process, from the number of records identified in the search to the number of studies included in the review, ideally using a flow diagram. | Pg.7; MS and Figure 1 |
|  | 16b | Cite studies that might appear to meet the inclusion criteria, but which were excluded, and explain why they were excluded. | NA |
| **Study characteristics** | 17 | Cite each included study and present its characteristics. | Pg.9; MS and Supp. Table 1 and 2 |
| **Risk of bias in studies** | 18 | Present assessments of risk of bias for each included study. | Pg.9; MS and Supp. Figure 10 |
| **Results of individual studies** | 19 | For all outcomes, present, for each study: (a) summary statistics for each group (where appropriate) and (b) an effect estimate and its precision (e.g., confidence/credible interval), ideally using structured tables or plots. | Pg.7-8; MS;Fig. 2-6; Supp. Fig. 1-7 |
| **Results of syntheses** | 20a | For each synthesis, briefly summarize the characteristics and risk of bias among contributing studies. | Pg.9; MS and Supp. Figure 10 |
|  | 20b | Present results of all statistical syntheses conducted. If meta-analysis was done, present for each the summary estimate and its precision (e.g., confidence/credible interval) and measures of statistical heterogeneity. If comparing groups, describe the direction of the effect. | Pg.7-9; MS;Fig. 2-6; Supp. Fig. 1-9 |
|  | 20c | Present results of all investigations of possible causes of heterogeneity among study results. | Pg. 7-8; MS and Supp. Figure 8 |
|  | 20d | Present results of all sensitivity analyses conducted to assess the robustness of the synthesized results. | Pg. 8-9; MS and Supp. Figure 8 |
| **Reporting biases** | 21 | Present assessments of risk of bias due to missing results (arising from reporting biases) for each synthesis assessed. | Pg. Pg.9; MS and Supp. Figure 11 |
| **Certainty of evidence** | 22 | Present assessments of certainty (or confidence) in the body of evidence for each outcome assessed. | NA |
| **DISCUSSION** |  |  |  |
| **Discussion** | 23a | Provide a general interpretation of the results in the context of other evidence. | Pg. 9; MS |
|  | 23b | Discuss any limitations of the evidence included in the review. | Pg. 11; MS |
|  | 23c | Discuss any limitations of the review processes used. | Pg. 13; MS |
|  | 23d | Discuss implications of the results for practice, policy, and future research. | Pg. 12-13; MS |
| **OTHER INFORMATION** |  |  |  |
| **Registration and protocol** | 24a | Provide registration information for the review, including register name and registration number, or state that the review was not registered. | CRD42024566070 |
|  | 24b | Indicate where the review protocol can be accessed, or state that a protocol was not prepared. | <https://www.crd.york.ac.uk/prospero/display_record.php?RecordID=566070> |
|  | 24c | Describe and explain any amendments to information provided at registration or in the protocol. | NA |
| **Support** | 25 | Describe sources of financial or non-financial support for the review, and the role of the funders or sponsors in the review. | None |
| **Competing interests** | 26 | Declare any competing interests of review authors. | Pg. 1;MS |
| **Availability of data, code and other materials** | 27 | Report which of the following are publicly available and where they can be found template data collection forms; data extracted from included studies; data used for all analyses; analytic code; any other materials used in the review. | NA |

^Abbreviations: MS, manuscript; supp., supplement.^

**Supplemental Methods 2. PRISMA Abstract Checklist**

| **Topic** | **No.** | **Item** | **Reported?** |
| --- | --- | --- | --- |
| **TITLE** |  |  |  |
| **Title** | 1 | Identify the report as a systematic review. | Yes |
| **BACKGROUND** |  |  |  |
| **Objectives** | 2 | Provide an explicit statement of the main objective(s) or question(s) the review addresses. | Yes |
| **METHODS** |  |  |  |
| **Eligibility criteria** | 3 | Specify the inclusion and exclusion criteria for the review. | Yes |
| **Information sources** | 4 | Specify the information sources (e.g., databases, registers) used to identify studies and the date when each was last searched. | Yes |
| **Risk of bias** | 5 | Specify the methods used to assess risk of bias in the included studies. | No |
| **Synthesis of results** | 6 | Specify the methods used to present and synthesize results. | Yes |
| **RESULTS** |  |  |  |
| **Included studies** | 7 | Give the total number of included studies and participants and summarize relevant characteristics of studies. | Yes |
| **Synthesis of results** | 8 | Present results for main outcomes, preferably indicating the number of included studies and participants for each. If meta-analysis was done, report the summary estimate and confidence/credible interval. If comparing groups, indicate the direction of the effect (i.e., which group is favored). | Yes |
| **DISCUSSION** |  |  |  |
| **Limitations of evidence** | 9 | Provide a brief summary of the limitations of the evidence included in the review (e.g., study risk of bias, inconsistency and imprecision). | No |
| **Interpretation** | 10 | Provide a general interpretation of the results and important implications. | No |
| **OTHER** |  |  |  |
| **Funding** | 11 | Specify the primary source of funding for the review. | No |
| **Registration** | 12 | Provide the register name and registration number. | No |

**Supplemental Methods 3. Details of the Search Strategy**

| **Search Strategy for each database** | |
| --- | --- |
| **Pubmed** | (empagliflozin OR "Sodium-glucose co-transporter 2 inhibitors" OR "Sodium-Glucose Transporter 2 Inhibitors"[Mesh] OR SGLT2i OR dapagliflozin OR canagliflozin) AND ( "myocardial infarction" OR "Myocardial Infarction"[Mesh] OR MI OR AMI) AND (randomized[Title/Abstract] OR randomised[Title/Abstract] OR random[Title/Abstract] OR randomly[Title/Abstract] OR RCT[Title/Abstract]) |
| **EMBASE** | ('empagliflozin'/exp OR '1 chloro 4 (1 glucopyranosyl) 2 [ [4 (3 oxolanyloxy) phenyl] methyl] benzene' OR '1 chloro 4 (1 glucopyranosyl) 2 [ [4 (tetrahydro 3 furanyloxy) phenyl] methyl] benzene' OR '1 chloro 4 (1 glucopyranosyl) 2 [4 (3 oxolanyloxy) benzyl] benzene' OR '1 chloro 4 (1 glucopyranosyl) 2 [4 (tetrahydro 3 furanyloxy) benzyl] benzene' OR '1 chloro 4 (glucopyranos 1 yl) 2 [ [4 (oxolan 3 yloxy) phenyl] methyl] benzene' OR '1 chloro 4 (glucopyranos 1 yl) 2 [ [4 (tetrahydrofuran 3 yloxy) phenyl] methyl] benzene' OR '1 chloro 4 (glucopyranos 1 yl) 2 [4 (oxolan 3 yloxy) benzyl] benzene' OR '1 chloro 4 (glucopyranos 1 yl) 2 [4 (tetrahydrofuran 3 yloxy) benzyl] benzene' OR '1, 5 anhydro 1 [4 chloro 3 [ [4 [ (3 oxolanyl) oxy] phenyl] methyl] phenyl] glucitol' OR '1, 5 anhydro 1 [4 chloro 3 [ [4 [ (oxolan 3 yl) oxy] phenyl] methyl] phenyl] glucitol' OR '1, 5 anhydro 1 [4 chloro 3 [ [4 [ (tetrahydro 3 furanyl) oxy] phenyl] methyl] phenyl] glucitol' OR '1, 5 anhydro 1 [4 chloro 3 [ [4 [ (tetrahydrofuran 3 yl) oxy] phenyl] methyl] phenyl] glucitol' OR '1, 5 anhydro 1 [4 chloro 3 [4 [ (tetrahydro 3 furyl) oxy] benzyl] phenyl] glucitol' OR '1, 5 anhydro 1 c [4 chloro 3 [ [4 [ (3 oxolanyl) oxy] phenyl] methyl] phenyl] dextro glucitol' OR '1, 5 anhydro 1 c [4 chloro 3 [ [4 [ (oxolan 3 yl) oxy] phenyl] methyl] phenyl] dextro glucitol' OR '1, 5 anhydro 1 c [4 chloro 3 [ [4 [ (tetrahydro 3 furanyl) oxy] phenyl] methyl] phenyl] dextro glucitol' OR '1, 5 anhydro 1 c [4 chloro 3 [ [4 [ (tetrahydrofuran 3 yl) oxy] phenyl] methyl] phenyl] dextro glucitol' OR '2 [4 chloro 3 [ [4 (3 oxolanyl) oxyphenyl] methyl] phenyl] 6 (hydroxymethyl) oxane 3, 4, 5 triol' OR '2 [4 chloro 3 [ [4 (oxolan 3 yl) oxyphenyl] methyl] phenyl] 6 (hydroxymethyl) oxane 3, 4, 5 triol' OR '2 [4 chloro 3 [ [4 (tetrahydro 3 furanyl) oxyphenyl] methyl] phenyl] 6 (hydroxymethyl) oxane 3, 4, 5 triol' OR '2 [4 chloro 3 [ [4 (tetrahydrofuran 3 yl) oxyphenyl] methyl] phenyl] 6 (hydroxymethyl) oxane 3, 4, 5 triol' OR '2 [4 chloro 3 [4 [ (3 oxolanyl) oxy] benzyl] phenyl] 6 (hydroxymethyl) tetrahydro 2h pyran 3, 4, 5 triol' OR '2 [4 chloro 3 [4 [ (oxolan 3 yl) oxy] benzyl] phenyl] 6 (hydroxymethyl) tetrahydro 2h pyran 3, 4, 5 triol' OR '2 [4 chloro 3 [4 [ (tetrahydro 3 furanyl) oxy] benzyl] phenyl] 6 (hydroxymethyl) tetrahydro 2h pyran 3, 4, 5 triol' OR '2 [4 chloro 3 [4 [ (tetrahydrofuran 3 yl) oxy] benzyl] phenyl] 6 (hydroxymethyl) tetrahydro 2h pyran 3, 4, 5 triol' OR 'bi 10773' OR 'bi10773' OR 'ckd 398' OR 'ckd398' OR 'empagliflozin' OR 'gibtulio' OR 'jardiance' OR 'oboravo' OR 'sodium glucose cotransporter inhibitor'/exp OR 'sodium dependent glucose cotransporter inhibitor' OR 'sodium glucose co-transporter inhibitor' OR 'sodium glucose cotransporter inhibitor' OR 'dapagliflozin'/exp OR '1 [4 chloro 3 (4 ethoxybenzyl) phenyl] 1 deoxy beta d glucopyranose' OR '1, 5 anhydro 1 c [4 chloro 3 [ (4 ethoxyphenyl) methyl] phenyl] dextro glucitol' OR '2 (3 (4 ethoxybenzyl) 4 chlorophenyl) 6 hydroxymethyltetrahydro 2h pyran 3, 4, 5 triol' OR '2 [4 chloro 3 (4 ethoxybenzyl) phenyl] 6 (hydroxymethyl) oxane 3, 4, 5 triol' OR '2 [4 chloro 3 [ (4 ethoxyphenyl) methyl] phenyl] 6 (hydroxymethyl) oxane 3, 4, 5 triol' OR 'andatang' OR 'bms 512148' OR 'bms512148' OR 'ckd 380' OR 'ckd380' OR 'dapagliflozin' OR 'dapagliflozin acetate' OR 'dapagliflozin propanediol' OR 'dapagliflozin propanediol monohydrate' OR 'dwp 16001' OR 'dwp16001' OR 'edistride' OR 'farxiga' OR 'forxiga' OR 'hgp 1602' OR 'hgp 1812' OR 'hgp1602' OR 'hgp1812' OR 'lyn 045' OR 'lyn045' OR 'oxra' OR 'canagliflozin'/exp OR '1 (glucopyranosyl) 4 methyl 3 [5 (4 fluorophenyl) 2 thienylmethyl] benzene' OR '1, 5 anhydro 1 c [3 [ [5 (4 fluorophenyl) 2 thienyl] methyl] 4 methylphenyl] d glucitol' OR '1, 5 anhydro 1 c [3 [ [5 (4 fluorophenyl) thiophen 2 yl] methyl] 4 methylphenyl] d glucitol' OR '1, 5 anhydro 1 c [3 [5 (4 fluorophenyl) 2 thenyl] 4 methylphenyl] glucitol' OR '2 [3 [ [5 (4 fluorophenyl) 2 thiophenyl] methyl] 4 methylphenyl] 6 (hydroxymethyl) 3, 4, 5 oxanetriol' OR '2 [3 [ [5 (4 fluorophenyl) thiophen 2 yl] methyl] 4 methylphenyl] 6 (hydroxymethyl) oxane 3, 4, 5 triol' OR '2 [3 [5 (4 fluorophenyl) 2 thiophenylmethyl] 4 methylphenyl] 6 (hydroxymethyl) tetrahydro 3, 4, 5 pyrantriol' OR '2 [3 [5 (4 fluorophenyl) thiophen 2 ylmethyl] 4 methylphenyl] 6 (hydroxymethyl) tetrahydropyran 3, 4, 5 triol' OR 'canagliflocin' OR 'canagliflozin' OR 'canagliflozin hemihydrate' OR 'canaglu' OR 'invokana' OR 'jnj 28431754' OR 'jnj28431754' OR 'sulisent' OR 'ta 7284' OR 'ta7284') AND ('heart function'/exp OR 'cardiac function' OR 'function, heart' OR 'heart function' OR 'heart muscle function' OR 'myocardial function' OR 'myocardium function') AND ('randomized controlled trial'/exp OR 'controlled trial, randomized' OR 'randomised controlled study' OR 'randomised controlled trial' OR 'randomized controlled study' OR 'randomized controlled trial' OR 'trial, randomized controlled') |
| **Cochrane Library** | (empagliflozin OR "Sodium-glucose co-transporter 2 inhibitors" OR SGLT2i OR dapagliflozin OR canagliflozin ) AND ("myocardial infarction" OR MI OR AMI) AND ("randomized controlled trial"[pt] OR "controlled clinical trial"[pt] OR randomized[ab] OR placebo[ab] OR "drug therapy"[sh] OR randomly[ab] OR trial[ab] OR groups[ab]) |

**Supplemental Methods 4. Definitions of Outcomes**

| **Outcome** | **Definition** |
| --- | --- |
| **Heart Failure**  **Hospitalization** | Hospital admission due to any heart failure related symptom, lab/imaging findings or physical signs. The hospitalization required to be reported by an investigator. Heart failure hospitalizations were also considered as a worsening in heart failure episodes. (1) |
| **All-Cause Mortality** | All cause mortality is defined as any cause of death in a targeted population over a period of time.(2) |
| **Major Adverse Cardiovascular**  **Event (MACE)** | MACE was defined as cardiovascular death (CV death), myocardial infarction (MI) or all cause stroke. ( ) |
| **Cardiovascular**  **(CV) Death** | CV deaths include those resulting from a myocardial infarction (MI), sudden cardiac death, heart failure (HF), stroke, cardiovascular procedures, cardiovascular hemorrhage, and other CV causes. (3) |
| **Stroke** | Stroke endpoint is defined as an acute episode of focal or global neurological dysfunction caused by brain, spinal cord, or retinal vascular injury as a result of hemorrhage or infarction. (3) |

**Supplemental Table 1. Eligibility Criteria Used by Study**

| **Study and year** | **Eligibility Criteria** |
| --- | --- |
| **EMBODY 2020**(4) | Inclusion Criteria: “(1) Adults (aged ≥ 20 years); (2) Glycemic condition ( Subjects appropriately diagnosed as T2DM by the Japanese guideline - Drug-naïve subjects or taking single anti-diabetic agent - T2DM patients who need to start or are possibly changing or adding an anti-diabetic agent); (3)Patients within 2–12 weeks after the onset of AMI, who can be discharged home”  Exclusion Criteria: “(1)Type 1 diabetes mellitus; (2) Persistent atrial fiblilation; (3) Insulin and glucagon-like peptide-1 analog user; (4) High dose of sulfonylurea (glimepiride > 2 mg, glibenclamide > 1.25 mg, glimicron > 40 mg);(5). HbA1c ≥ 10%;(6.) History of diabetic ketoacidosis or diabetic coma within 3 months prior to the randomization;(7) Renal dysfunction (eGFR < 45 ml/min/1.73 m²);(8) Heart failure graded at NYHA functional class IV;(9) Pregnancy or possible pregnancy and breastfeeding; (10) Lack of informed consent; and (11) Contraindications to empagliflozin according to the label”  *Study Protocol, Table 1.* |
| **DECLARE–TIMI 58 2018**(5)      **DECLARE–TIMI 58 2018**  ***(continued)***  **DECLARE–TIMI 58 2018**  ***(continued)*** | Inclusion Criteria: “For inclusion in the study patients should fulfill the following criteria:(1) Provision of informed consent prior to any study specific procedures (including run-in);(2) Female or male aged ≥ 40 years;(3) Diagnosed with T2DM (See Appendix E for details); (4) High Risk for CV event defined as having either established CV disease and/or multiple risk factors:(a)Established CV Disease (See Appendix E for details) OR (b)No known cardiovascular disease AND at least two cardiovascular risk factors in addition toT2DM, defined as:Age > 55 years in men and > 60 in women; AND presence of at least 1 of the following additional risk factors:(i) Dyslipidemia; (ii)Hypertension; (iii)Current Tobacco use. (5) WOCBP must take precautions to avoid pregnancy throughout the study and for 4 weeks after intake of the last dose.(a) WOCBP must have a negative urine pregnancy test. WOCBP include any female who has experienced menarche and who has not undergone successful surgical sterilization (hysterectomy, bilateral tubal ligation or bilateral oophorectomy) or is not postmenopausal.(b)WOCBP must be willing to use a medically accepted method of contraception that is considered reliable in the judgment of the Investigator.”  Exclusion Criteria:(1) Use of the following excluded medications:(a)Current or recent (within 24 months) treatment with pioglitazone and/or use of pioglitazone for a total of 2 years or more during lifetime;(b)Current or recent (within 12 months) treatment with rosiglitazone;(c)Previous treatment with any SGLT2 inhibitor;(d)Any patient currently receiving chronic (>30 consecutive days) treatment with an oral steroid at a dose equivalent to oral prednisolone ≥10 mg (e.g., betamethasone ≥1.2 mg,dexamethasone ≥1.5 mg, hydrocortisone ≥40 mg) per day. (2) Acute cardiovascular event[e.g., acute coronary syndrome (ACS), transient ischemic attack (TIA), stroke, any revascularization, decompensated HF, sustained ventricular tachycardia <8 weeks prior to randomization. Patients with acute cardiovascular events can be enrolled in the run-in period as long as randomization does not occur within 8 weeks of the event.(3) Systolic BP >180 or diastolic BP >100 mmHg at randomization. Patient should be excluded if either the systolic BP is elevated (> 180 mmHg) or the diastolic BP is elevated (> 100 mmHg) on both measurements (see section 6.4.8.1).(4) Diagnosis of Type 1 diabetes mellitus, MODY, or secondary diabetes mellitus.(5) History of bladder cancer or history of radiation therapy to the lower abdomen or pelvis at any time.(6) History of any other malignancy within 5 years (with the exception of successfully treated non-melanoma skin cancers).(7) Chronic cystitis and/or recurrent urinary tract infections (3 or more in the last year).(8) Any conditions that, in the opinion of the Investigator, may render the patient unable to complete the study including but not limited to cardiovascular (NYHA class IV, CHF, recurrent ventricular arrhythmias) or non-cardiovascular disease (e.g., active malignancy with the exception of basal cell carcinoma, cirrhosis, chronic lung disease, severe autoimmune disease) and/or a likely fatal outcome within 5 years.(9) Pregnant or breast-feeding patients. (10) Involvement in the planning and/or conduct of the study or other dapagliflozin studies(applies to AZ, BMS, Hadassah and Thrombolysis in Myocardial Infarction [TIMI] or representative staff and/or staff at the study site).(11) Previous enrollment or randomization in the present study.(12) Active participation in another clinical study with IP and/or investigational device.(13) Individuals at risk for poor protocol or medication compliance during run-in period(reasonable compliance defined as 80 – 120%, unless a reason for non-compliance is judged acceptable by the Investigator). If for any reason, the Investigator believes that the patient will not tolerate or be compliant with IP or study procedures, the patient should not be randomized and considered a run-in failure.Patients will be excluded during run-in and should not be randomized if the following are observed from laboratory or observation during enrollment and run-in assessments(14) HbA1c ≥12% or HbA1c<6.5% from the central laboratory (nb, the proportion of subjects with an HbA1c between 6.5 % and < 7.0 % will be capped at approximately 5% of the study);(15) AST or ALT >3x ULN or Total bilirubin >2.5 x ULN;(16). CrCl < 60 ml/min (based on the Cockroft-Gault equation).(17) Hematuria (confirmed by microscopy at Visit 1) with no explanation as judged by the Investigator up to randomization. If bladder cancer is identified, patients are not eligible to participate.(18) Any reason the Investigator believes the patient is not likely to be compliant with the study medication and protocol.”  *Study Protocol, Pages 37 to 39* |
| **Adel et al. 2022**(6) | Inclusion criteria: “included:(1) age over 18 years and previous diagnosis of diabetes mellitus (fasting blood sugar [FBS] ≥126 mg/dL;(2)oral glucose tolerance test ≥200 mg/dL;(3)hemoglobin A1C [HbA1C] ≥6.5%, (4) classic symptoms of hyperglycemia with BS ≥200 mg/dL) with ACS (ST elevation myocardial infarction [MI], (5)Non-STelevation MI [STEMI],(6) unstable angina).”  Exclusion criteria: “In addition,(1) patients with diabetic ketoacidosis,(2) urinary and genital infections,(3) type 1 diabetes, (4) severe liver failure,(5) any malignancy and cancer,(6) glomerular filtration rate (eGFR) <30 mL/min/1.73m2, and (6) non-adherence to treatment procedure were excluded from the study.”  *Full Text; pag.2; Methods Section* |
| **EMPACT-MI 2024**(7)  **EMPACT-MI 2024**  ***(continued)*** | Inclusion criteria: Included: (1) Of full age of consent (according to local legislation, at least ≥ 18 years) at screening; (2) signed and dated written informed consent in accordance with ICH-GCP and local legislation prior to admission to the trial; (3) male or female patients. Women of childbearing potential (WOCBP) must be ready and able to use highly effective methods of birth control per ICH M3 (R2) that result in a low failure rate of less than 1% per year when used consistently and correctly. A list of contraception methods meeting these criteria is provided in the patient information and in the protocol; (4) diagnosis of spontaneous AMI: STEMI or NSTEMI with randomisation to occur no later than 14 calendar days after hospital admission. For patients with an in-hospital MI as qualifying event,randomization must still occur within 14 days of hospital admission; (5)High risk of HF, defined as EITHER(a) Symptoms (e.g. dyspnea; decreased exercise tolerance; fatigue), or signs of congestion (e.g.pulmonary rales, crackles or crepitations; elevated jugular venous pressure; congestion on chest X-ray), that require treatment (e.g. augmentation or initiation of oral diuretic therapy; i.v. diuretic therapy;i.v. vasoactive agent; mechanical intervention etc.) at any time during the hospitalization.OR(b) Newly developed LVEF < 45% as measured by echocardiography, ventriculography, cardiac CT,MRI or radionuclide imaging during index hospitalization.(6) In addition at least one of the following risk factors:(a) Age ≥ 65 years;(b) Newly developed LVEF < 35%;(b) Prior MI (before index MI) documented in medical records;(c)eGFR < 60 ml/min/1.73m2 (using CKD-EPI formula based on creatinine from local lab at any time during index hospitalization);(d) Atrial fibrillation (persistent or permanent; if paroxysmal, only valid if associated with index MI);(e) Type 2 diabetes mellitus (prior or new diagnosis);(f) NT-proBNP ≥1,400 pg/mL for patients in sinus rhythm, ≥2,800 pg/mL if atrial fibrillation; BNP ≥350 pg/mL for patients in sinus rhythm, ≥700 pg/mL if atrial fibrillation, measured at any time during hospitalization,(g) Uric acid ≥7.5 mg/dL (≥446 μmol/L), measured at any time during hospitalization;(h) Pulmonary Artery Systolic Pressure [or right ventricular systolic pressure] ≥40 mmHg (non-invasive[usually obtained from clinically indicated post-MI echocardiography] or invasive, at any time during hospitalization);(i)Patient not revascularized (and no planned revascularization) for the index MI (Includes e.g. patients where no angiography is performed, unsuccessful revascularization attempts, diffuse atherosclerosis not amenable for intervention; but does NOT include if revascularization was not performed due to nonobstructive coronary arteries),3-vessel coronary artery disease at time of index MI and diagnosis of peripheral artery disease (extracoronary vascular disease, e.g. lower extremity artery disease or carotid artery disease).”  Exclusion criteria: “(1) diagnosis of chronic HF prior to index MI; (2) Systolic blood pressure ≤ 90 mmHg at randomisation; (3) cardiogenic shock or use of i.v. inotropes in last 24 hours before randomisation; (4) coronary Artery Bypass Grafting planned at time of randomisation; (5) current diagnosis of Takotsubo cardiomyopathy; (6) Any current severe (stenotic or regurgitant) valvular heart disease; (7)eGFR < 20 ml/min/1.73m2 (using CKD-EPI formula based on most recent creatinine from local lab during index hospitalization) or on dialysis; (8) type I diabetes mellitus; (9) History of ketoacidosis; (10) Current use or planned treatment with an SGLT-2 inhibitor or combined SGLT-1 and 2 inhibitor. Discontinuation of a SGLT-2 inhibitor or combined SGLT-1 and 2 inhibitor for the purposes of enrollment in the trial is not permitted; (11) contraindication for using empagliflozin or any other SGLT-2 inhibitor; (12) any physical or mental condition significantly affecting the patient’s ability to participate in the investigator’s opinion; (13) Any other clinical condition that would jeopardise patient’s safety while participating in this study, or may prevent the patient from adhering to the trial protocol in the Investigator’s opinion; (14) presence of any other disease than the acute MI or its immediate complications with a life expectancy of <1 year in the opinion of the investigator; (15) current or previous randomisation in one of the empagliflozin heart failure trials (i.e. trials 1245.110,1245.121, 1245-0167, 1245-0168, 1245-0204) or currently enrolled in another investigational device or drug trial, or less than 30 days since ending another investigational device or drug trial, or receiving other investigational treatment(s). Patients participating in purely observational trial will not be excluded; (16) Women who are pregnant, nursing, or who plan to become pregnant while in the trial. "  *Study Protocol; Pages 3 to 5* |
| **DACAMI 2023**(8) | Inclusion Criteria:“(1)Patients admitted with criteria for anterior ST- elevation myocardial infarction according to the fourth universal definition of myocardial infarction; (2)show echocardiographic evidence of reduced LV ejection fraction <50%; and (3)have undergone successful reperfusion by primary percutaneous coronary angiography (pPCI).”  Exclusion Criteria:“(1) Patients with Diabetes Mellitus Type 2 (T2DM), Type 1 (T1DM), secondary diabetes (e.g., endocrinopathies) (2) Patients already diagnosed with heart failure before this event (3) Patients on cardiotoxic chemotherapeutic medications. (4) Patients with hemoglobinopathies. (5) Patients with chronic organ damage (i.e., chronic hepatitis with MELD score > 10, Stage 4 & 5 renal disease). (6) Patients already on SGLT2i. (7) Patients requiring additional anticoagulant therapy (i.e., patients with transthoracic echocardiographic evidence of left ventricular thrombus). (8) Patients with contraindications for the use of dapagliflozin, including patients with severely impaired renal function (eGFR <30 ml/min/1.73 m2) &/OR previous history of genitourinary infections (i.e.,urosepsis, pyelonephritis & Fournier's gangrene) &/OR at high risk of such infections.”  *Full Text; pag.2; Methods Section* |
| **DAPA MI 2023**(9)  **DAPA MI 2023**  ***(continued)*** | Inclusion criteria: " (1) Men or women age ≥18 at the time of signing the informed consent; (2) confirmed myocardial infarction (MI), either ST elevation MI or non-ST-elevation MI, according to the fourth universal definition of MI, 2 within the preceding 7 days, or 10 days if earlier randomization is not feasible; (3) imaging evidence of impaired regional or global left ventricular (LV) systolic function at any timepoint during the index MI-related hospitalization (established with echocardiogram, radionuclide ventriculogram, contrast angiography or cardiac magnetic resonance imaging) OR definitive evidence on electrocardiogram of a Q-wave MI (defined as presence of Q waves in two or more contiguous leads, excluding leads III and aVR, and meeting all the following criteria: at least 1.5 mm in depth; at least 30 ms in duration; and, if R wave present, more than 25% of the size of the subsequent R wave); (4) hemodynamically stable at randomization (no episodes of symptomatic hypotension, or arrhythmia with hemodynamic compromise in the last 24 hours); (5) capable of giving signed informed consent that includes compliance with the requirements and restrictions listed in the informed consent form and in the protocol; (6) provision of signed and dated, written informed consent prior to any mandatory trial specific procedures, sampling, and analyses"  Exclusion criteria: " (1) known type 1 or type 2 diabetes at the time of admission. Patients with hyperglycemia, but without a diagnosis of diabetes mellitus prior to the index event, were eligible at the discretion of the investigator; (2) chronic symptomatic heart failure (HF) with a prior hospitalization due to HF within the last year and known reduced LV ejection fraction (LVEF≤40 %), documented before the current MI hospitalization; (3) severe chronic kidney disease (eGFR <20 mL/min/1.73 m2 by local laboratory), unstable or rapidly progressing kidney disease at the time of recruitment; (4) severe hepatic impairment (Child-Pugh class C) at the time of recruitment for the trial; (5) active malignancy requiring treatment at the time of screening, except for basal cell- or squamous cell carcinoma of the skin, presumed possible to treat successfully; (6) any non-CV condition, e.g. malignancy, with a life expectancy of less than two years based on the investigator ́s clinical judgment; (7) currently on treatment, or with an indication for treatment, with a sodium glucose co-transporter 2 inhibitor (SGLT2-inhibitor); (8) known intolerance to dapagliflozin; (9) participation in a)another trial with a non-approved investigational drug or blinded treatment with a CV or glucose lowering medication b)the planning and/or conduct of the trial (applies to AstraZeneca staff, Uppsala Clinical Research staff, and/or staff at the trial site) c) previous randomization in the present trial (10) judgment by the investigator that the participant should not participate in the trial if the participant is unlikely to comply with trial procedures, restrictions and requirements, or any condition in the opinion of the Investigator that would make participation unsafe or unsuitable; (11) women of childbearing potential (i.e., those who are not chemically or surgically sterilized or postmenopausal): a) Who are not willing to use a highly effective method of contraception, OR b)Who have a positive pregnancy test, OR c)Who are breast-feeding"  *Supplementary material; Table S1* |
| **CANVAS 2021**(10)  **CANVAS 2021**  **(continued)**  **CANVAS 2021**  **(continued)**  **CANVAS 2021**  **(continued)**  **CANVAS 2021**  **(continued)** | Inclusion criteria were: " (1) man or woman with a diagnosis of type 2 diabetes with glycated hemoglobin level ≥7.0% to ≤10.5% at screening and be either (a) not currently on antihyperglycemic agent (AHA) therapy or (b) on AHA monotherapy or combination therapy with any approved class of agents: e.g., sulfonylurea, metformin, peroxisome proliferator-activated receptor gamma (PPARγ) agonist, alpha-glucosidase inhibitor, glucagon-like peptide-1 (GLP-1) analogue, dipeptidyl peptidase-4 (DPP-4) inhibitor, or insulin; (2) History or high risk of cardiovascular disease defined on the basis of either:  –Age ≥30 years with documented symptomatic atherosclerotic cardiovascular disease: including stroke; myocardial infarction (MI); hospital admission for unstable angina; coronary artery bypass graft (CABG); percutaneous coronary intervention (PCI; with or without stenting); peripheral revascularization (angioplasty or surgery); symptomatic with documented hemodynamically-significant carotid or peripheral vascular disease; or amputation secondary to vascular disease.  – Age ≥50 years with 2 or more of the following risk factors determined at the screening visit: duration of type 2 diabetes of 10 years or more, systolic blood pressure >140 mmHg (average of 3 readings) recorded at the screening visit, while the subject is on at least one blood pressure–lowering treatment, current daily cigarette smoker, documented microalbuminuria or macroalbuminuria, or documented high-density lipoprotein (HDL)  cholesterol of <1 mmol/l (<39 mg/dl);  (3) women must be:  – Postmenopausal, defined as >45 years of age with amenorrhea for at least 18 months, or  >45 years of age with amenorrhea for at least 6 months and less than 18 months and a  serum follicle stimulating hormone (FSH) level >40 IU/ml, or  – Surgically sterile (have had a hysterectomy or bilateral oophorectomy, tubal ligation), or otherwise be incapable of pregnancy, or  – Heterosexually active and practicing a highly effective method of birth control, including  hormonal prescription oral contraceptives, contraceptive injections, contraceptive patch, intrauterine device, double-barrier method (e.g., condoms, diaphragm, or cervical cap with spermicidal foam, cream, or gel), or male partner sterilization, consistent with local  regulations regarding use of birth control methods for subjects participating in clinical trials, for the duration of their participation in the study, or  – Not heterosexually active.  Note: subjects who are not heterosexually active at screening must agree to utilize a highly effective method of birth control if they become heterosexually active during their participation in the study; (4) women of childbearing potential must have a negative urine β-human chorionic gonadotropin (β-hCG) pregnancy test at screening and baseline (predose, Day 1); (5) willing and able to adhere to the prohibitions and restrictions specified in this protocol; (6) subjects must have signed an informed consent document indicating that they understand the purpose of and procedures required for the study and are willing to participate in the study; (7) to participate in the optional pharmacogenomic component of this study, subjects must have signed the informed consent form for pharmacogenomic research indicating willingness to participate in the pharmacogenomic component of the study (where local regulations permit). Refusal to give consent for this component does not exclude a subject from participation in the clinical study; (8) subjects must have taken ≥80% of their single-blind placebo capsules during the 2-week run-in period at Day 1 to be eligible for randomization.  Exclusion criteria were: " (1) history of diabetic ketoacidosis, type 1 diabetes, pancreas or beta-cell transplantation, or diabetes secondary to pancreatitis or pancreatectomy; (2) on an AHA and not on a stable regimen (i.e., agents and doses) for at least 8 weeks before  the screening visit and through the screening/run-in period. Note: a stable dose of insulin  is defined as no change in the insulin regimen (i.e., type[s] of insulin) and ≤15% change in the total daily dose of insulin (averaged over 1 week to account for day-to-day variability); (3) fasting fingerstick glucose at site >270 mg/dl (>15 mmol/l) at Baseline/Day 1  – For patients on a sulfonylurea agent or on insulin: fasting fingerstick glucose at site  <110 mg/dl (<6 mmol/l) at Baseline/Day 1.  Note: at the investigator’s discretion, based upon an assessment of recent self-monitored  blood glucose (SMBG) values, subjects meeting either of these fingerstick glucose exclusion  criteria may continue the single-blind placebo and return to the investigational site within  14 days and may be randomized if the repeat fasting fingerstick value no longer meets the  exclusion criterion. Subjects with fingerstick glucose >270 mg/dl (>15 mmol/l) may have  their AHA regimen adjusted and be rescreened once on a stable regimen for at least 8 weeks; (4) history of one or more severe hypoglycemic episode within 6 months before screening.  Note: a severe hypoglycemic episode is defined as an event that requires the help of another person; (5) history of hereditary glucose-galactose malabsorption or primary renal glucosuria; (6) ongoing, inadequately controlled thyroid disorder.  Note: subjects on thyroid hormone-replacement therapy must be on a stable dose for at least 6 weeks before Day 1; (7) renal disease that required treatment with immunosuppressive therapy or a history of dialysis or renal transplant.  Note: subjects with a history of treated childhood renal disease, without sequelae, may participate; (8) MI, unstable angina, revascularization procedure, or cerebrovascular accident within 3 months before screening, or a planned revascularization procedure, or history of New York  Heart Association (NYHA) Class IV cardiac disease; (9) findings on 12-lead electrocardiogram (ECG) that would require urgent diagnostic evaluation of intervention (e.g., new clinically important arrhythmia or conduction  disturbance); (10) known ECG findings within 3 months  before screening that would require urgent diagnostic evaluation or intervention (e.g., new clinically important arrhythmia or conduction disturbance); (11) history of hepatitis B surface antigen or hepatitis C antibody positive (unless associated with documented persistently stable/normal range aspartate aminotransferase [AST] and  alanine aminotransferase [ALT] levels), or other clinically active liver disease; (12) any history of or planned bariatric surgery; (13) estimated glomerular filtration rate (eGFR) <30 ml/min/1.73 m2 at screening (provided by the central laboratory)  – For subjects taking metformin: at screening, serum creatinine ≥1.4 mg/dl (124 μmol/l) for  men or ≥1.3 mg/dl (115 μmol/l) for women; no contraindication to the use of metformin (including eGFR) based on the label of the country of investigational site  eGFR <30 ml/min/1.73 m2 at screening visit; (14) ALT levels >2.0 times the upper limit of normal (ULN) or total bilirubin >1.5 times the ULN at screening, unless in the opinion of the investigator and as agreed upon by the sponsor’s medical officer, the findings are consistent with Gilbert’s disease; ( 15) history of malignancy within 5 years before screening (exceptions: squamous and basal cell  carcinomas of the skin and carcinoma of the cervix in situ, or a malignancy that in the opinion of the investigator, with concurrence with the sponsor’s medical monitor, is considered cured with minimal risk of recurrence);  (16) history of human immunodeficiency virus (HIV) antibody positive; (17) subject has a current clinically important hematological disorder (e.g., symptomatic anemia, proliferative bone marrow disorder, thrombocytopenia); (18) investigator’s assessment that the subject’s life expectancy is less than 1 year, or any  condition that in the opinion of the investigator would make participation not in the best interest of the subject, or could prevent, limit, or confound the protocol-specified safety or  efficacy assessments; (19) major surgery (i.e., requiring general anesthesia) within 3 months of the screening visit or  any surgery planned during the subject’s expected participation in the study (except minor surgery; i.e., outpatient surgery under local anesthesia); (20) any condition that, in the opinion of the investigator, would compromise the well-being of the subject or prevent the subject from meeting or performing study requirements; (21) prior or current participation in another canagliflozin study; (22) current use of other sodium glucose co-transporter 2 (SGLT2) inhibitor. Current or prior use of an SGLT2 inhibitor; (23) known allergies, hypersensitivity, or intolerance to canagliflozin or its excipients; (24) current use of a corticosteroid medication or immunosuppressive agent, or likely to require treatment with a corticosteroid medication (for longer than 2 weeks in duration) or an  immunosuppressive agent. Note: subjects using inhaled, intranasal, intra-articular, or topical corticosteroids, or corticosteroids in therapeutic replacement doses may participate; (25) received an active investigational drug (including vaccines) or used an investigational  medical device within 3 months before Day 1/baseline or received at least one dose of canagliflozin in a prior study; (26) history of drug or alcohol abuse within 3 years before screening; (27) pregnant or breastfeeding or planning to become pregnant or breastfeed during the study;  (28) employees of the investigator or study center, with direct involvement in the proposed study or other studies under the direction of that investigator or study center, as well as family members of the employees or the investigator. |
| **EMPA-REG**  **OUTCOME 2018**(11)  **EMPA-REG**  **OUTCOME 2018**  ***(continued)*** | Inclusion Criteria:“(1)Diagnosis of type 2 diabetes mellitus prior to informed consent; (2) Male or female patients on diet and exercise regimen who are drug naive or pre-treated with any background therapy. Antidiabetic therapy has to be unchanged for 12 weeks prior to randomization.;(3) Glycosylated haemoglobin (HbA1c) of >= 7.0% and <=10% for patients on background therapy or HbA1c >= 7.0% and <= 9.0% for drug naive patients; (4)Age >= 18 years; (5)Body Mass index <= 45 at Visit 1;(6)Signed and dated informed consent and (6)High cardiovascular risk”  Exclusion Criteria:“(1)Uncontrolled hyperglycemia with a glucose level >240 mg/dl (>13.3 mmol/L) after an overnight fast during placebo run-in and confirmed by a second measurement (not on the same day);(2)Indication of liver disease, defined by serum levels of either alanine aminotransferase (ALT), aspartate aminotransferase ALT or alkaline phosphatase above 3 x upper limit of normal (ULN) as determined at screening and/or run in;(3)Planned cardiac surgery or angioplasty within 3 months; (4)Impaired renal function, defined as Glomerular Filtration Rate <30 ml/min (severe renal impairment, Modification of Diet in Renal Disease formula) during screening or run in; (5)Bariatric surgery within the past two years and other gastrointestinal surgeries that induce chronic malabsorption;(6)Blood dyscrasias or any disorders causing haemolysis or unstable Red Blood Cell (e.g. malaria, babesiosis, haemolytic anemia);(7)Medical history of cancer (except for basal cell carcinoma) and/or treatment for cancer within the last 5 years;(8)Contraindications to background therapy according to the local label;(9)Treatment with anti-obesity drugs (e.g. sibutramine, orlistat) 3 months prior to informed consent or any other treatment at the time of screening (i.e. surgery, aggressive diet regimen, etc.) leading to unstable body weight;(10)Current treatment with systemic steroids at time of informed consent or change in dosage of thyroid hormones within 6 weeks prior to informed consent or any other uncontrolled endocrine disorder except type 2 diabetes mellitus; (11)Pre-menopausal women (last menstruation <+ 1 year prior to informed consent) who:are nursing or pregnant or are of child-bearing potential and are not practicing an acceptable method of birth control, or do not plan to continue using this method throughout the study and do not agree to submit to periodic pregnancy testing during participation in the trial. Acceptable methods of birth control include tubal ligation, transdermal patch, intrauterine devices/systems, oral, implantable or injectable contraceptives, sexual abstinence, double barrier method and vasectomised partner; (12)Alcohol or drug abuse within the 3 months prior to informed consent that would interfere with trial participation or any ongoing condition leading to a decreased compliance to study procedures or study drug intake; (13)Participation in another trial with an investigational drug within 30 days prior to informed consent; (14)Any other clinical condition that would jeopardize patients safety while participating in this clinical trial; and (15) Acute coronary syndrome, stroke or TIA within 2 months prior to informed consent.”  *Supplementary Material, Section D; Pages 18 and 19* |
| **EMMY 2022**(12)  **EMMY 2022**  ***(continued)*** | Inclusion Criteria: “(1) Myocardial infarction with evidence of significant myocardial necrosis defined as a rise in creatinine kinase >800 U/l and a troponin T-level (or troponin I-level) >10x ULN. In addition, at least 1 of the following criteria must be the met:(a) Symptoms of ischemia.;(b)ECG changes indicative of new ischemia (new ST-T changes or new LBBB)and (c) Imaging evidence of new regional wall motion abnormality.(2) 18 – 80 years of age.(3) Informed consent has to be given in written form.(4) eGFR > 45 ml/min/1.73m2.(5) Blood pressure before first drug dosing: systolic blood pressure>110mmHg.(6) Blood pressure before first drug dosing: diastolic blood pressure>70mmHg.(7) First intake of study medication ≤72h after myocardial infarction after performance of a coronary angiography”  Exclusion Criteria: “(1) Any other form of diabetes mellitus than type 2 diabetes mellitus, history of diabetic ketoacidosis.(2) Blood pH < 7,32.(3) Known allergy to SGLT-2 inhibitors.(4) Haemodynamic instability as defined by intravenous administration of catecholamine,calcium sensitizers or phosphodiesterase inhibitors.(5) >1 episode of severe hypoglycaemia within the last 6 months under treatment with insulin or sulfonylurea.(6) Females of child bearing potential without adequate contraceptive methods (i.e.sterilisation, intrauterine device, vasectomised partner; or medical history of hysterectomy).(7) Acute symptomatic urinary tract infection (UTI) or genital infection.(8) Patients currently being treated with any SGLT-2 inhibitor (dapagliflozin, canagliflozin,empagliflozin) or having received treatment with any SGLT-2 inhibitor within the 4 weeks prior to the screening visit.”  *Supplementary Material; Pages 9 and 10* |
| **DELIVERY 2022**(13) | Inclusion Criteria: “Briefly, (1) patients with or without diabetes were required to be (2) 40 years of age or older, (3)with an LVEF >40% (documented by echocardiography or cardiac magnetic resonance imaging within the last 12 months prior to enrolment without a subsequent event that might lower LVEF), (4)evidence of structural heart disease (either left atrial enlargement or left ventricular hypertrophy), and (5) elevation in natriuretic peptides [N-terminal pro B-type natriuretic peptide (NT-proBNP) ≥300 pg/mL(≥600 pg/mL for patients in atrial fibrillation or flutter)]. (6)Both ambulatory and hospitalized patients were eligible for enrolment.”  Exclusion Criteria: “Key exclusion criteria included (1)receiving an SGLT2 inhibitor within 4 weeks prior to randomization, or previous intolerance to SGLT2 inhibitors; (2)type 1 diabetes; (3)estimated glomerular filtration rate (eGFR) <25 mL/min/1.73 m2 at screening; (4)systolic blood pressure ≥160 mmHg if not on three or more antihypertensive medications, or ≥180 mmHg regardless of number of medications; (5) probable alternative diagnoses that might account for the patients’ symptoms (e.g. anaemia, hypothyroidism, primary pulmonary hypertension, chronic thromboembolic disease, requirement for home oxygen therapy); (6)uncorrected primary valvular disease; (7)known infiltrative heart disease, including known or suspected amyloid heart disease; myo- or pericarditis; or hypertrophic cardiomyopathy.”  *Study Protocol; Page 2; Patients Section;* |
| **DAPA-HF 2019**(13)  **DAPA-HF 2019**  ***(continued)*** | Inclusion Criteria: “For inclusion in the study patients should fulfill the following criteria:(1) Provision of signed informed consent prior to any study specific procedures; (2) Male or female, aged 218 years at the time of consent; (3) Established documented diagnosis of symptomatic HFrEF (New York Heart Association (NYHA) functional class II-I), which has been present for at least 2 months and is optimally treated with pharmacological and/or device therapy, as indicated NB: Patients in which additional pharmacological or device therapy is contemplated, or should be considered, must not be enrolled until therapy has been optimized and is stable for 21 month; (4)Left ventricular ejection fraction (LVEF) 54% (echocardiogram, radionuclide ventriculogram, contrast angiography or cardiac MRI) within the last 12 months prior to enrolment (Vist 1):(a) If There is more than one assessment of LVer the value from the most recent measurement should be used in assessing eligibility. (b) Patients undergoing coronary revascularization (percutaneous coronary intervention (PCI) or coronary artery bypass grafting (CABG)), valve repair/replacement or implantation of a cardiac resynchronization therapy (CRT) device or any other surgical, device or pharmacological intervention (ie initiation of a beta-blocker) that might improve LVEF must have a measurement of LVEF at least 3 months after the intervention in order to be eligible. NB: Patients with known HFrEF but without a recent (12 months) assessment of left ventricular (LV) function will undergo a local echocardiogram at the time of enrolment; (5) N-terminal pro b-type natriuretic peptide (NT-proBNP) >600 pg/mI (or if hospitalized for heart failure within the previous 12 months, NT-proBNP 2400 pg/mi) at enrolment (visit 1). If concomitant atrial fibrillation or atrial flutter at Visit 1, NT-proBNP must be ≥900 pg/mI(irrespective of history of heart failure hospitalization); (6) Patients should receive background standard of care for HrEF and be treated according to locally recognized guidelines with both drugs and devices, as appropriate. Guideline-recommended medications should be used at recommended doses unless contraindicated or not tolerated. Therapy should have been individually optimized and stable for 24 weeks (this does not apply to diuretics - see NB below) beforevisit 1 and include (unless contraindicated or not tolerated): (a) an ACE inhibitor, or ARB or sacubitril/valsartan and (b) a beta-blocker and c) if considered appropriate by the patient's treating physician, a mineralocorticoid receptor antagonist (MRA) NB: Most patients with heart failure require treatment with a diuretic to control sodium and water retention leading to volume overload. It is recognized that diuretic dosing may be titrated to symptoms, signs, weight and other information and may thus vary. Each patient should, however, be treated with a diuretic regimen aimed at achieving optimal fluid/volume status for that individual.(7) eGFR 230 ml/min/1.73 m2 (CKD-EPI formula) at enrolment (visit 1)”  Exclusion Criteria: “Key exclusion criteria included (1)receiving an SGLT2 inhibitor within 4 weeks prior to randomization, or previous intolerance to SGLT2 inhibitors; (2)type 1 diabetes; (3)estimated glomerular filtration rate (eGFR) <25 mL/min/1.73 m2 at screening; (4)systolic blood pressure ≥160 mmHg if not on three or more antihypertensive medications, or ≥180 mmHg regardless of number of medications; (5) probable alternative diagnoses that might account for the patients’ symptoms (e.g. anaemia, hypothyroidism, primary pulmonary hypertension, chronic thromboembolic disease, requirement for home oxygen therapy); (6)uncorrected primary valvular disease; (7)known infiltrative heart disease, including known or suspected amyloid heart disease; myo- or pericarditis; or hypertrophic cardiomyopathy.”  *Full Text; Page 2; Patients Section* |

**Supplemental Table 2. Additional Studies Characteristics**

| **Study and**  **year** | **Country** | **Previous MI** | **Previous Stroke** | **BMI**  **(kg/m2)** | **Smoking** | **Hypertension** |
| --- | --- | --- | --- | --- | --- | --- |
| **DAPA MI**  **2023** | United Kingdom and Sweden | 367 (9.13) | 96 (2.38) | N/A | 2301 (57.28) | 1482 (36.89) |
| **Adel et al. 2022** | Iran | N/A | N/A | N/A | 17 (18.27) | 58 (60.41) |
| **EMBODY 2020** | Japan | N/A | N/A | 25.2 | Current: 51 (53.12) | 77 (80.20) |
| **EMMY**  **2022** | Austria | 23 (4.8) | 6 (1.3) | 27.6 | 341 (72) | 199 (42) |
| **EMPACT-MI**  **2024** | North America,  Latin America,  Europe and Asia | 847 (13) | N/A | 28.1 | N/A | 4538 (69.58) |
| **EMPAREG OUTCOME**  **2019** | (North America ,Latin America, Europe, Africa, or Asia). | N/A | N/A | N/A | N/A | N/A |
| **DECLARE TIMI 58 2018** | 33 countries | 3584 | 218 (6.1) | N/A | 554 (15.5) | 3130 (87.3) |
| **DACAMI 2023** | Egypt | N/A | 0 | N/A | 66 (66%) | 61 (61%) |
| **DELIVERY + DAPA-HF 2024** | South America, Asia/Pacific, Europe, Saudi Arabia, and North America | 3731(100) | 441 (11.8) | 28.6 | Current: 503 (13.5) \| Former: 1810 (48.5) | 3114 (83.5) |
| **CANVAS 2021** | 30+ countries | N/A | N/A | 32.0 | 1806 (17.8) | 9125 (90.0) |

Binary data is displayed as a number (%). Median (min-max).* Data reported from entire study population, not only myocardial infarction patients.SGLT2: sodium-glucose co-transporter 2; T2DM: type 2 diabetes; eGFR: estimated glomerular filtration rate; STEMI: ST-elevation myocardial infarction. NA: not applicable

**Supplemental Figure 1. Forest Plot for Time to Event Heart Failure Hospitalization**


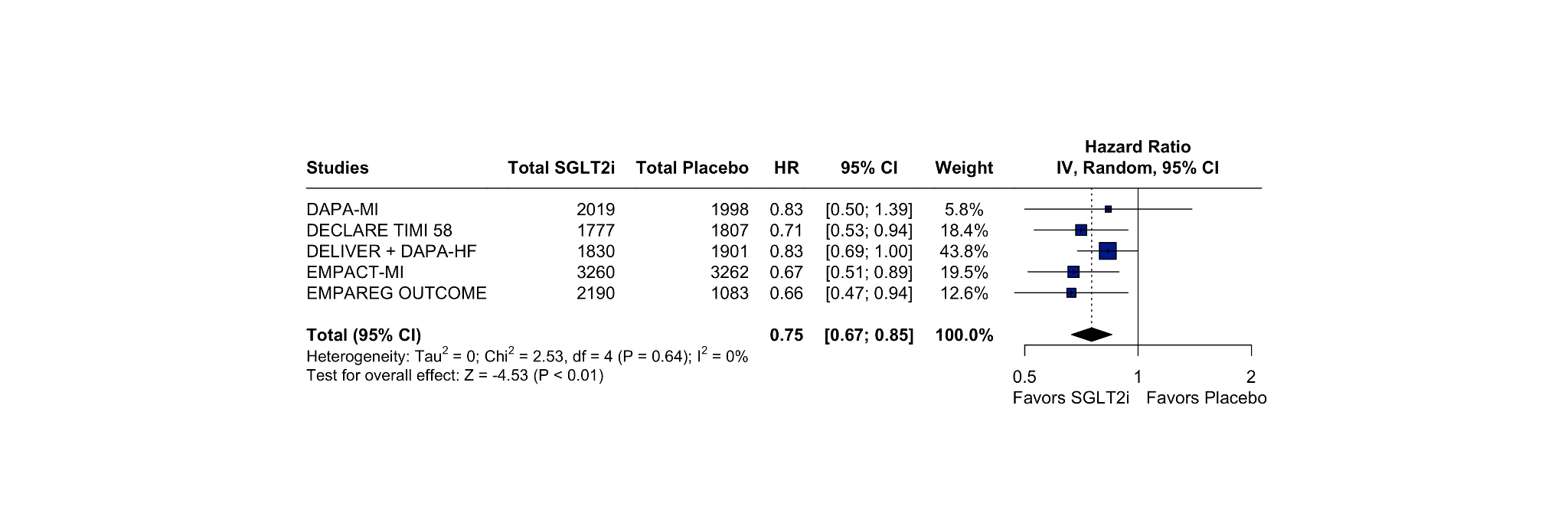


*Legend:* Patients with MI events using SGLT-2 had a significant decrease in risk for HF hospitalization compared to placebo in a time to event analysis. *Abbreviations:* CI: Confidence Interval;HF: Heart Failure; HR: Hazard Ratio; IV: Inverse Variance; MI: Myocardial infarction; SGLT2i: Sodium-glucose-transporter-2 inhibitors

**Supplemental Figure 2. Forest Plot for Time to All-Cause Mortality**


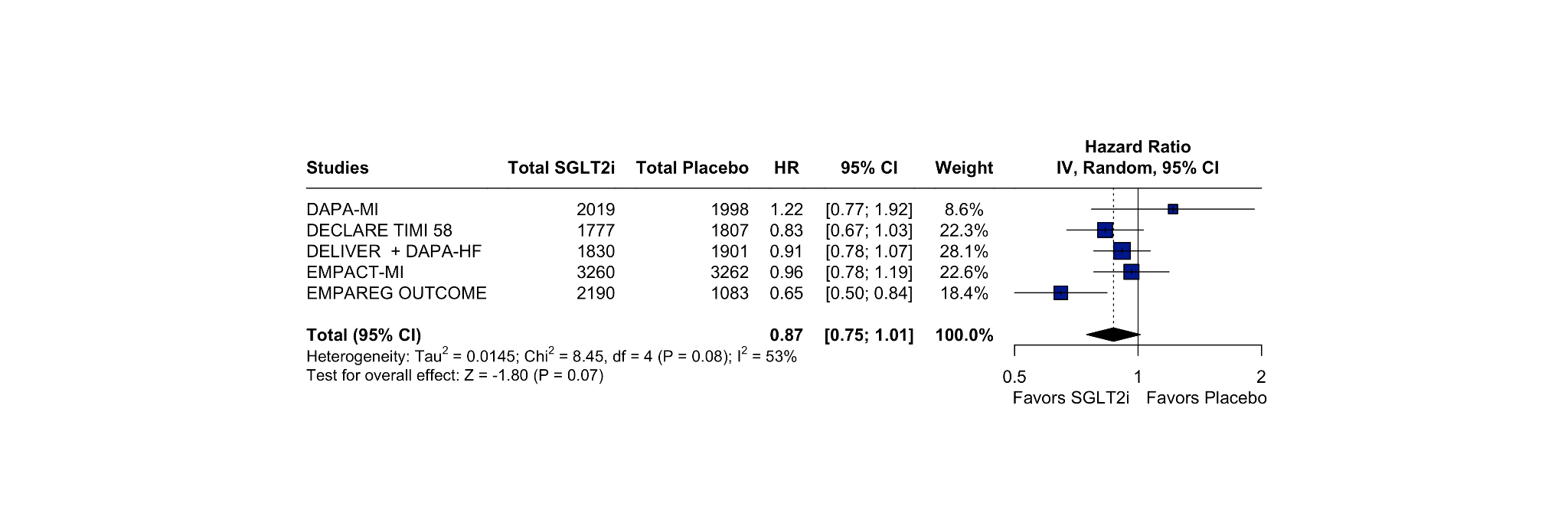


*Legend:* Patients with MI events using SGLT-2 had a significant decrease in risk for MI recurrence compared to placebo in a time to event analysis. *Abbreviations:* CI: Confidence Interval; HR: Hazard Ratio; IV: Inverse Variance; MI: Myocardial infarction; SGLT2i: Sodium-glucose-transporter-2 inhibitors

**Supplemental Figure 3. Forest Plot for Time to Event Major Adverse Cardiovascular Events

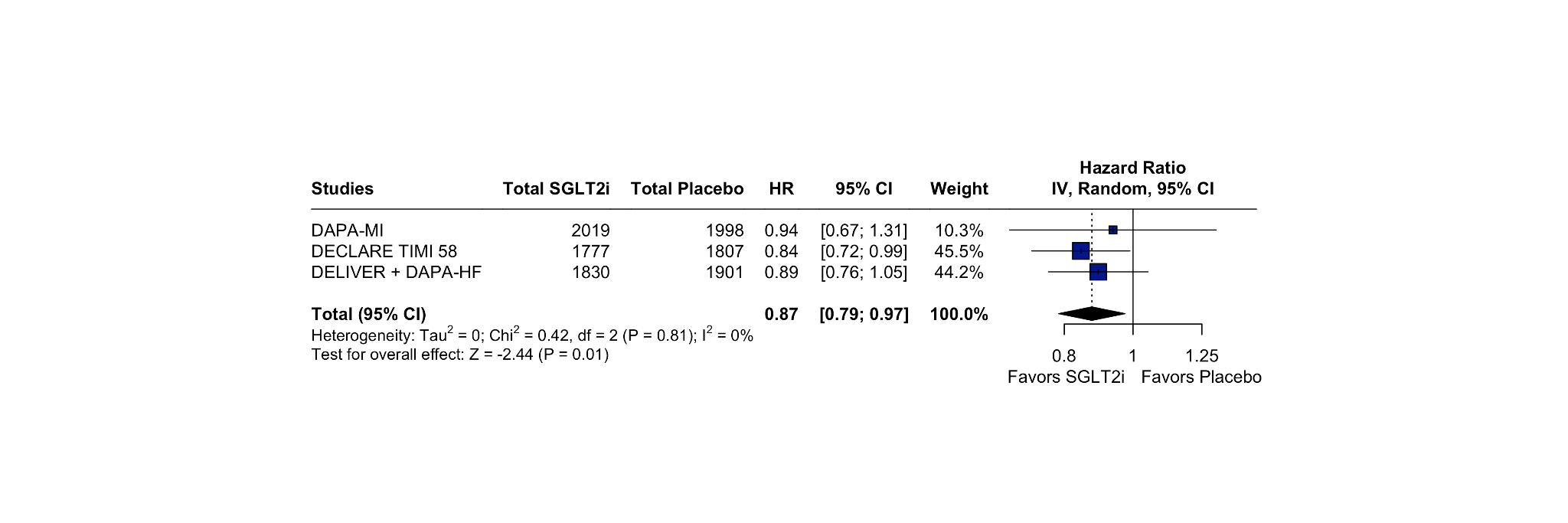
**

*Legend:* Patients with MI events using SGLT-2 had a significant risk decrease in MACE endpoint compared to placebo in a time to event analysis. *Abbreviations:* CI: Confidence Interval; HR: Hazard Ratio; IV: Inverse Variance; MACE: Major Adverse Cardiovascular Events; MI: Myocardial infarction; SGLT2i: Sodium-glucose-transporter-2 inhibitors

**Supplemental Figure 4. Forest Plot for Myocardial Infarction Endpoint**

**Figure 4A.** Forest Plot for Risk of MI Recurrence

**
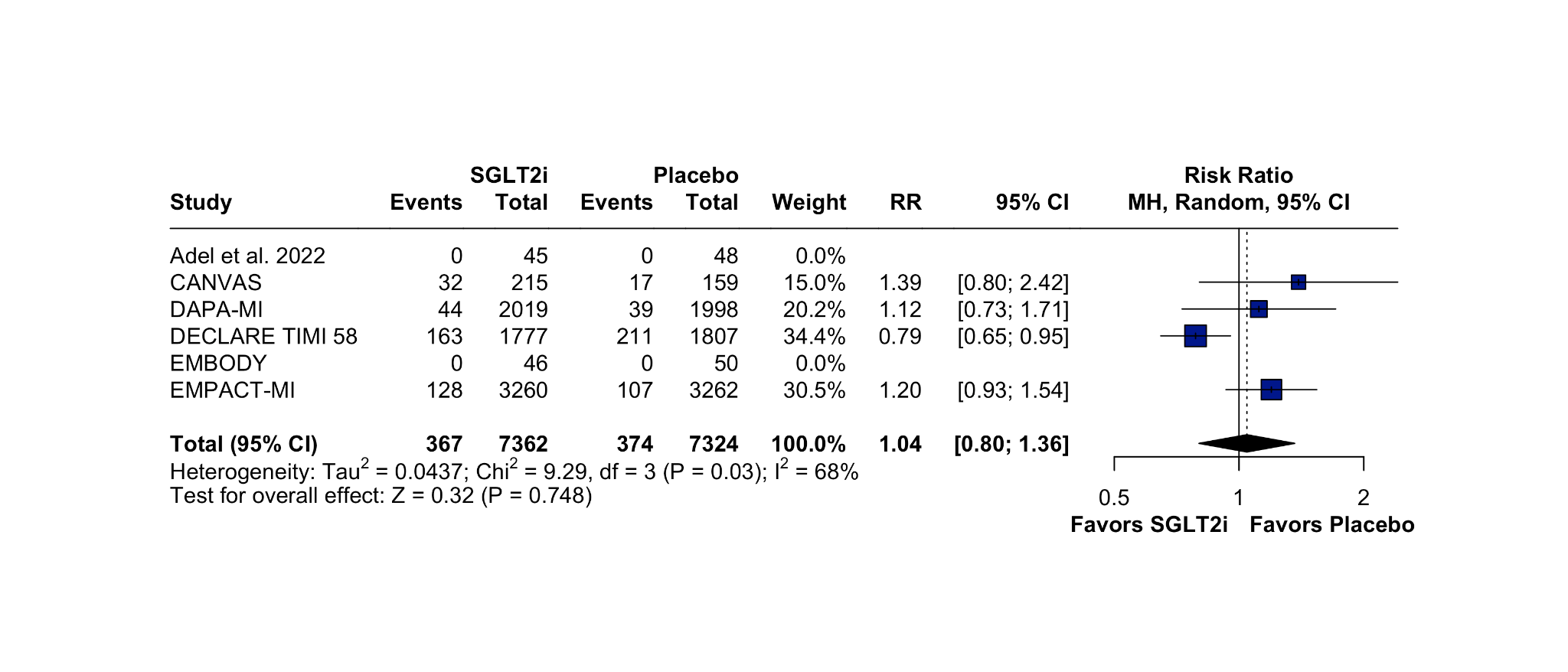
**

*Legend:* Patients with MI events using SGLT-2 had a non-significant decrease in risk for MI recurrence endpoint compared to placebo. *Abbreviations:* CI: Confidence Interval; MH: Mantel-Haenszel; MI: Myocardial infarction; RR: Risk Ratio; SGLT2i: Sodium-glucose-transporter-2 inhibitors

**Figure 4B.** Forest Plot for Time to Event of MI Recurrence


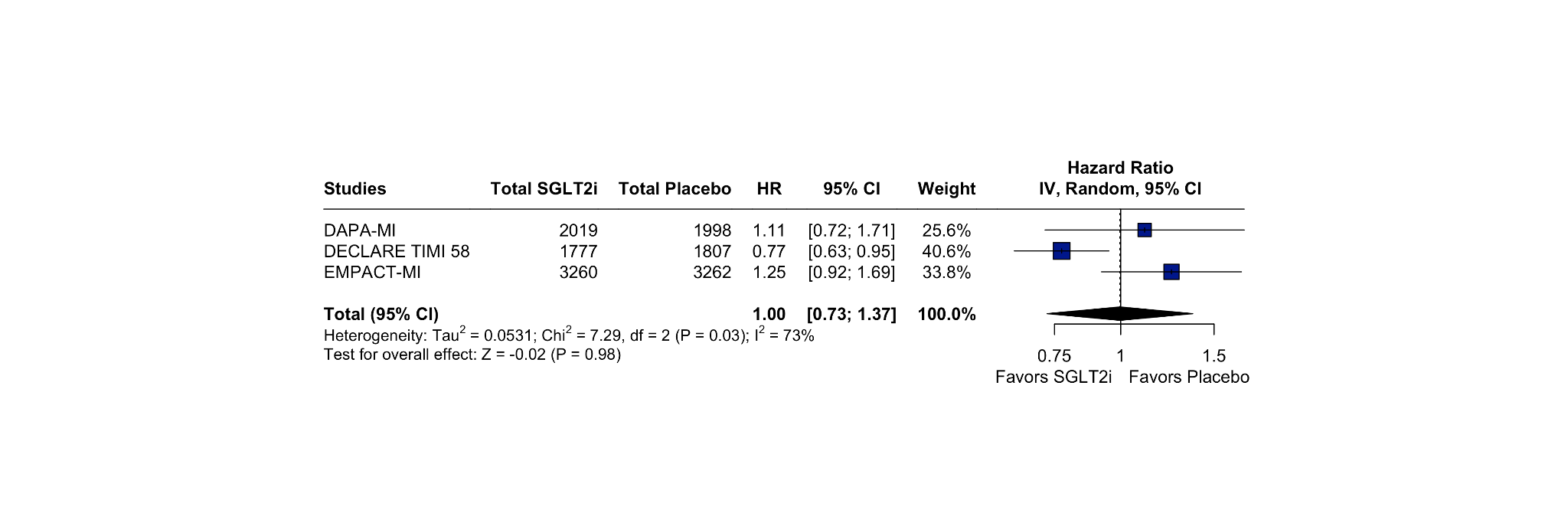


*Legend:* Patients with MI events using SGLT-2 had a non-significant decrease in risk for MI recurrence compared to placebo in a time to event analysis. *Abbreviations:* CI: Confidence Interval; HR: Hazard Ratio; IV: Inverse Variance; MI: Myocardial infarction; SGLT2i: Sodium-glucose-transporter-2 inhibitors

**Supplemental Figure 5. Forest Plot for Cardiovascular Death Endpoint**

**Figure 5A.** Forest Plot for risk of CV Death


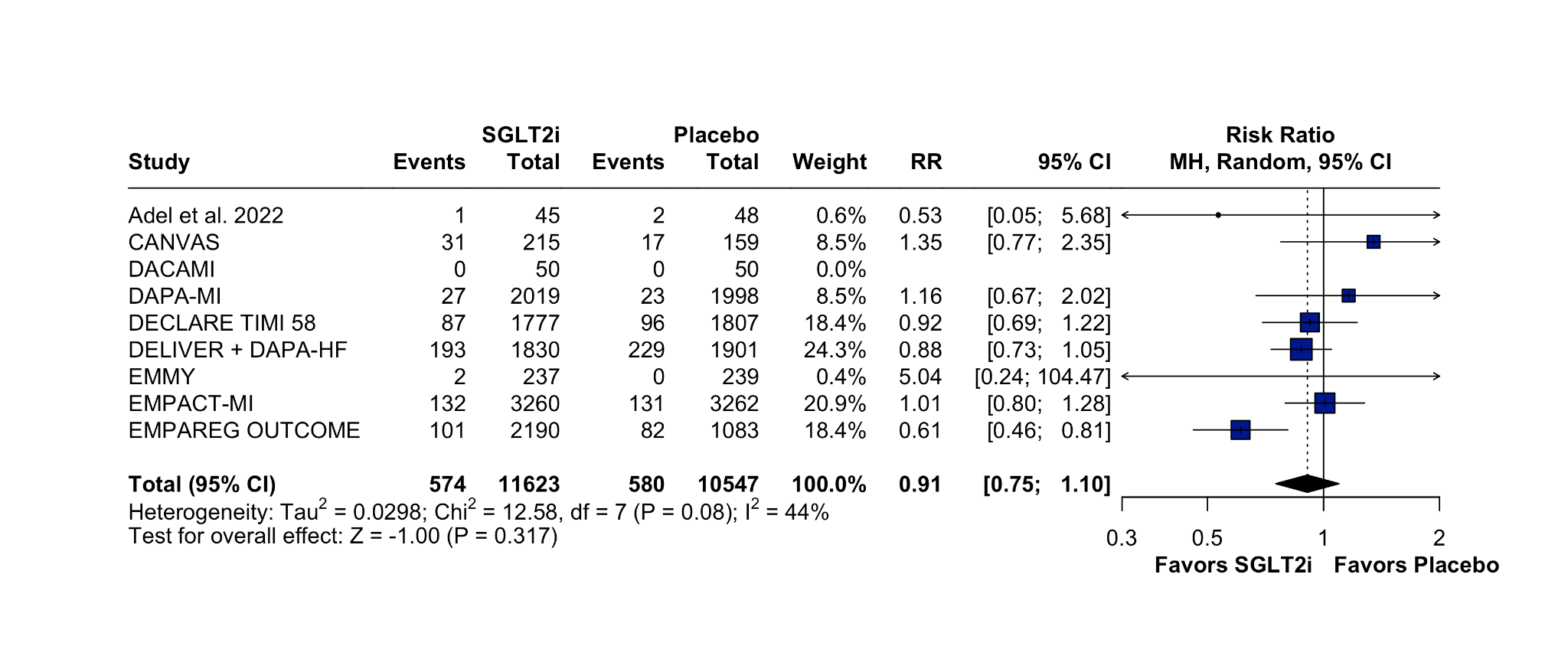


*Legend:* Patients with MI events using SGLT-2 had a non-significant decrease in risk for CV Death endpoint compared to placebo. *Abbreviations:* CI: Confidence Interval; CV: Cardiovascular; MH: Mantel-Haenszel; MI: Myocardial infarction; RR: Risk Ratio; SGLT2i: Sodium-glucose-transporter-2 inhibitors

**Figure 5B.** Forest Plot for Time to Event of CV Death


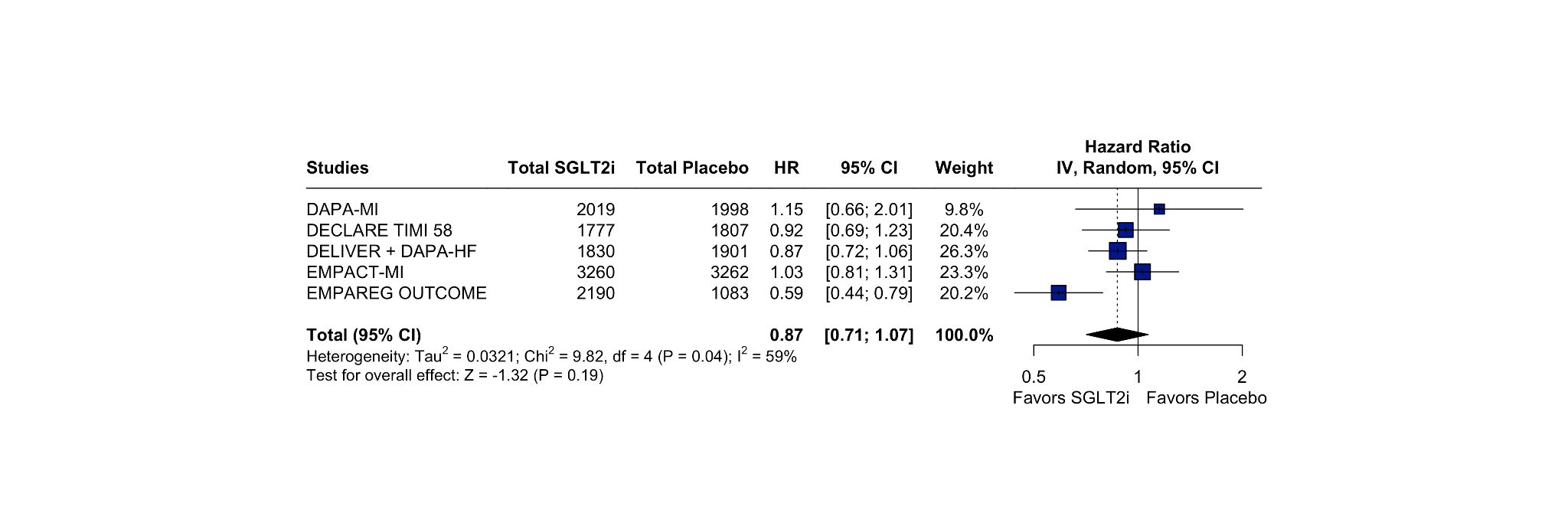


*Legend:* Patients with MI events using SGLT-2 had a non-significant decrease in risk for CV Death endpoint compared to placebo in a time to event analysis. *Abbreviations:* CI: Confidence Interval;Cardiovascular; HR: Hazard Ratio; IV: Inverse Variance; MI: Myocardial infarction; SGLT2i: Sodium-glucose-transporter-2 inhibitors

**Supplemental Figure 6. Forest Plot for Stroke Endpoint**
**Figure 6A.** Forest Plot for risk of Stroke


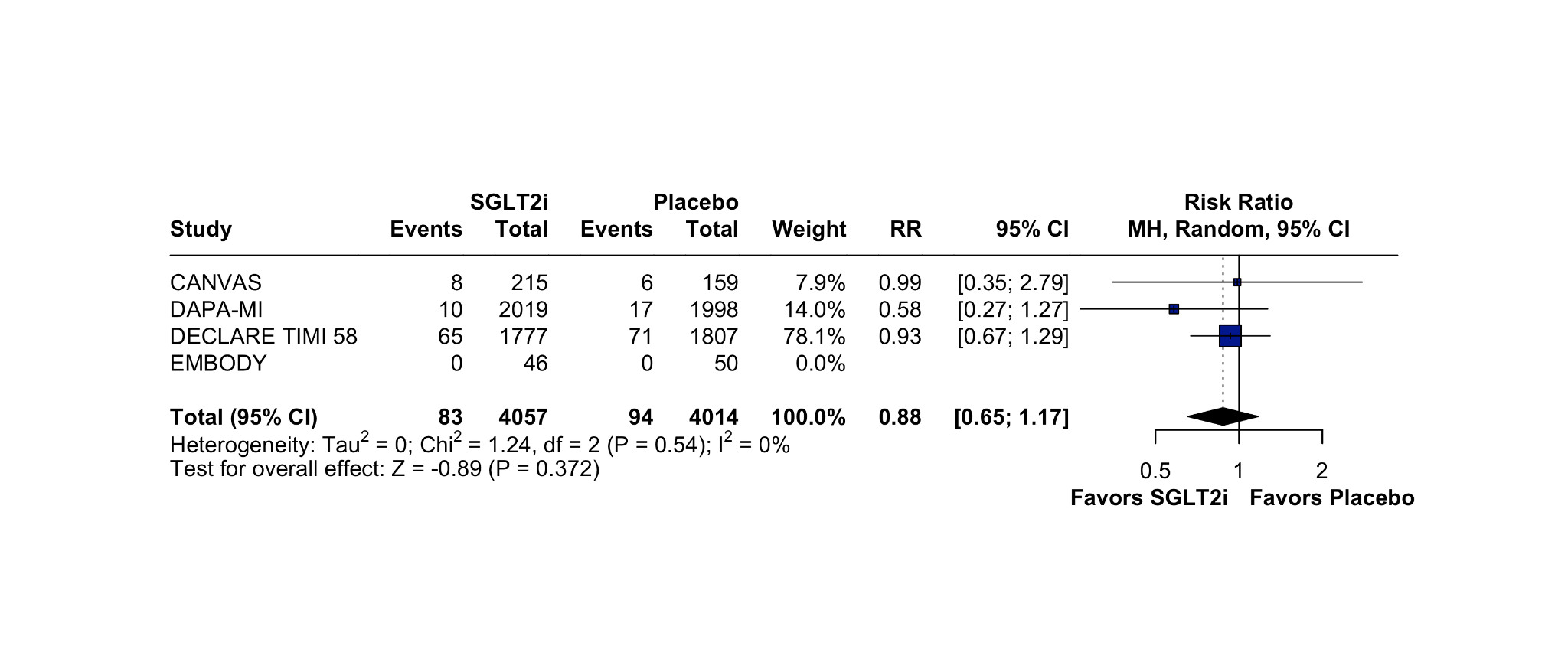


*Legend:* Patients with MI events using SGLT-2 had a non-significant decrease in stroke endpoint compared to placebo. *Abbreviations:* CI: Confidence Interval; MH: Mantel-Haenszel; MI: Myocardial infarction; RR: Risk Ratio; SGLT2i: Sodium-glucose-transporter-2 inhibitors

**Figure 6B.** Forest Plot for Time to Event of Stroke


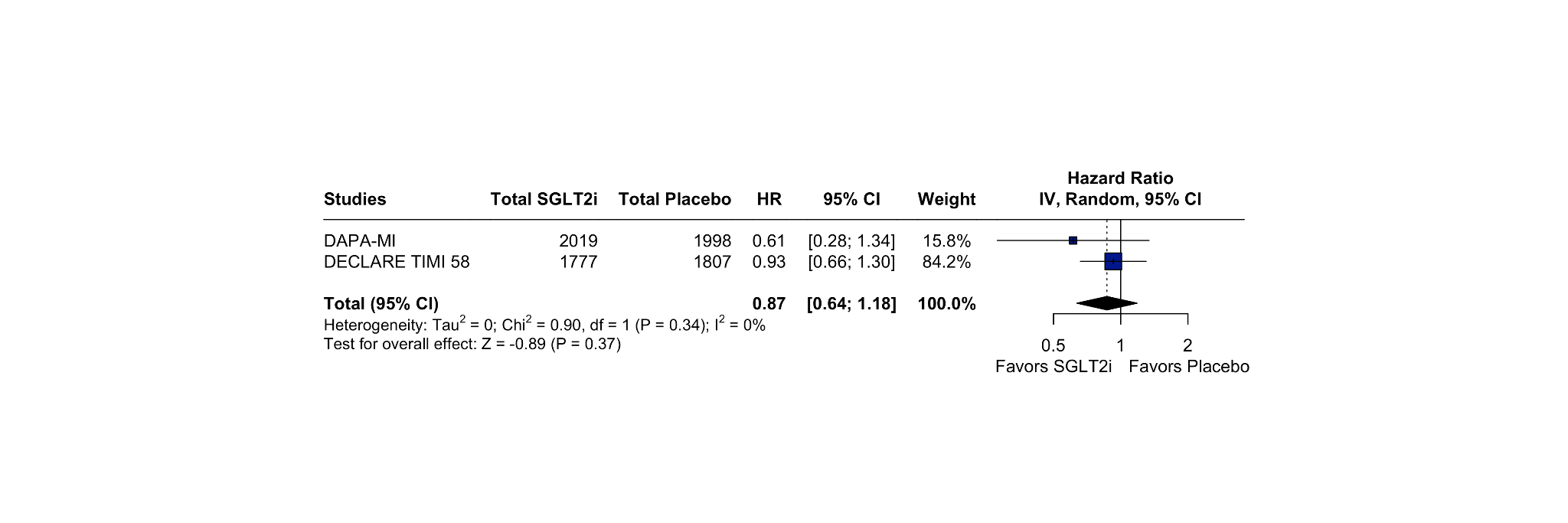


*Legend:* Patients with MI events using SGLT-2 had a non-significant decrease in stroke endpoint compared to placebo in a time to event analysis. *Abbreviations:* CI: Confidence Interval; HR: Hazard Ratio; IV: Inverse Variance; MI: Myocardial infarction; SGLT2i: Sodium-glucose-transporter-2 inhibitors

**Supplemental Figure 7. Forest Plot for Subgroup Analysis for Empagliflozin versus Dapagliflozin**


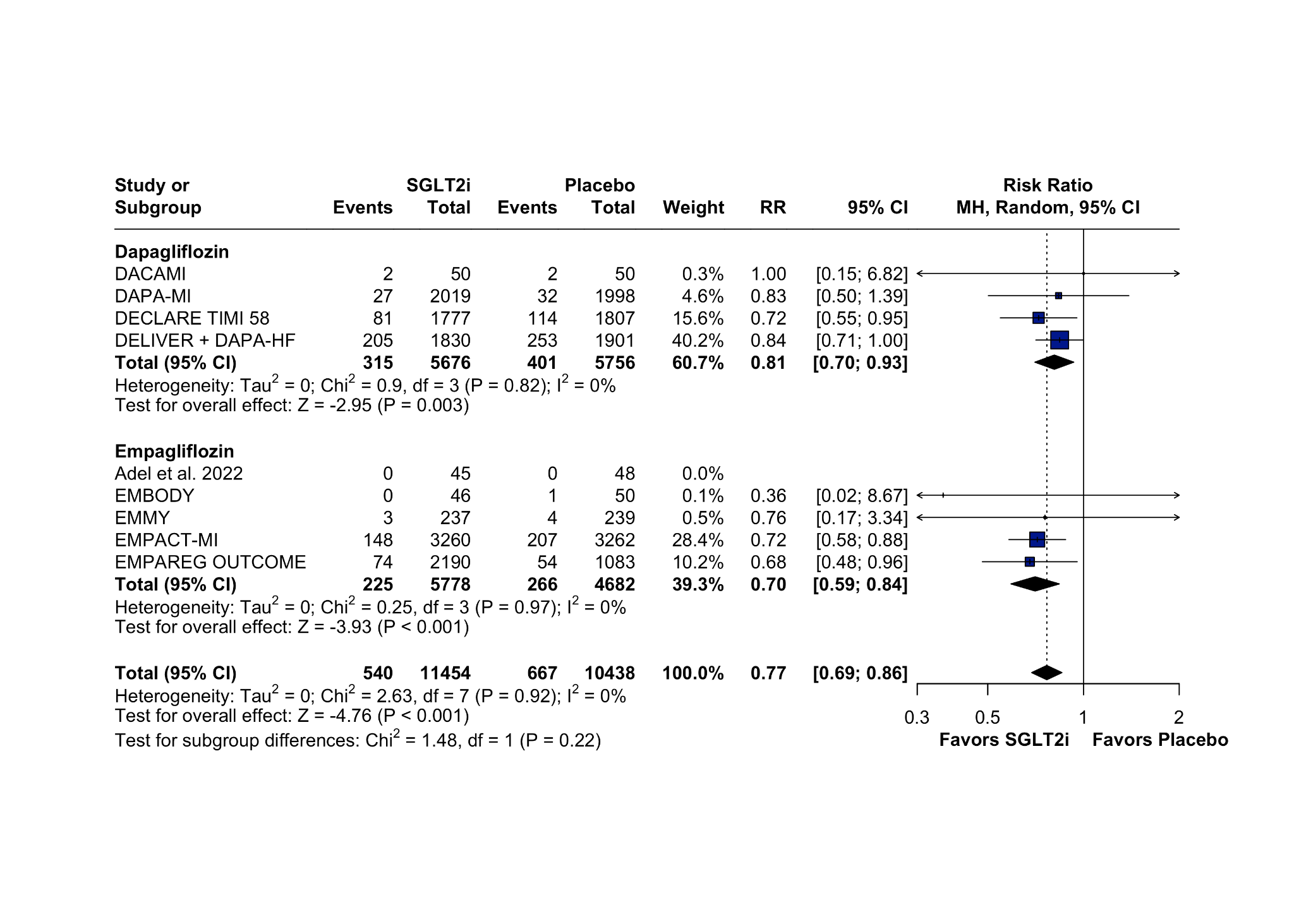


*Legend: Empagliflozin and Dapagliflozin had similar efficacy in reducing HF hospitalizations compared to placebo*. Subgroups *Abbreviations:* CI: Confidence Interval; MH: Mantel-Haenszel; MI: Myocardial infarction; RR: Risk Ratio; SGLT2i: Sodium-glucose-transporter-2 inhibitors

**Supplemental Figure 8. Sensitivity Analyses**
**Figure 8A**. Leave one out analysis for HF Hospitalization


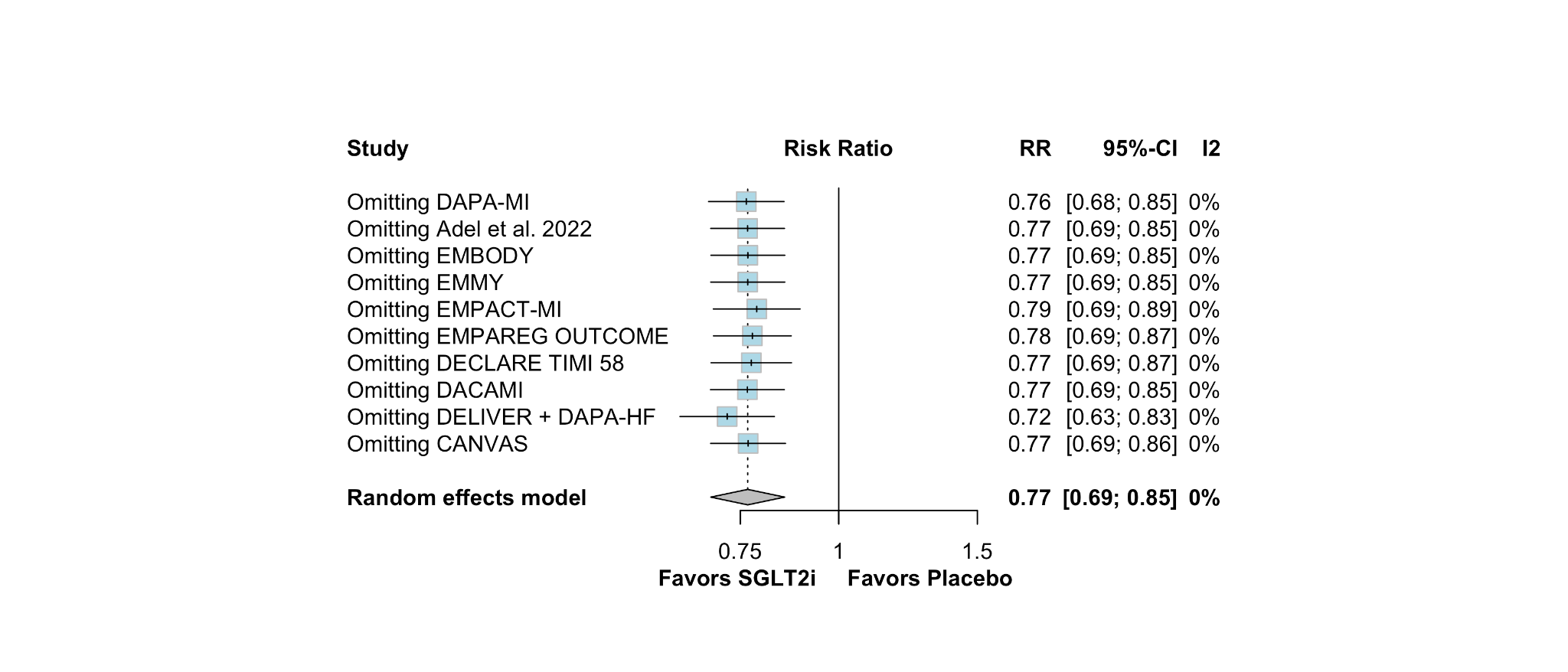


**Figure 8B.** Leave one out analysis for All-Cause Mortality


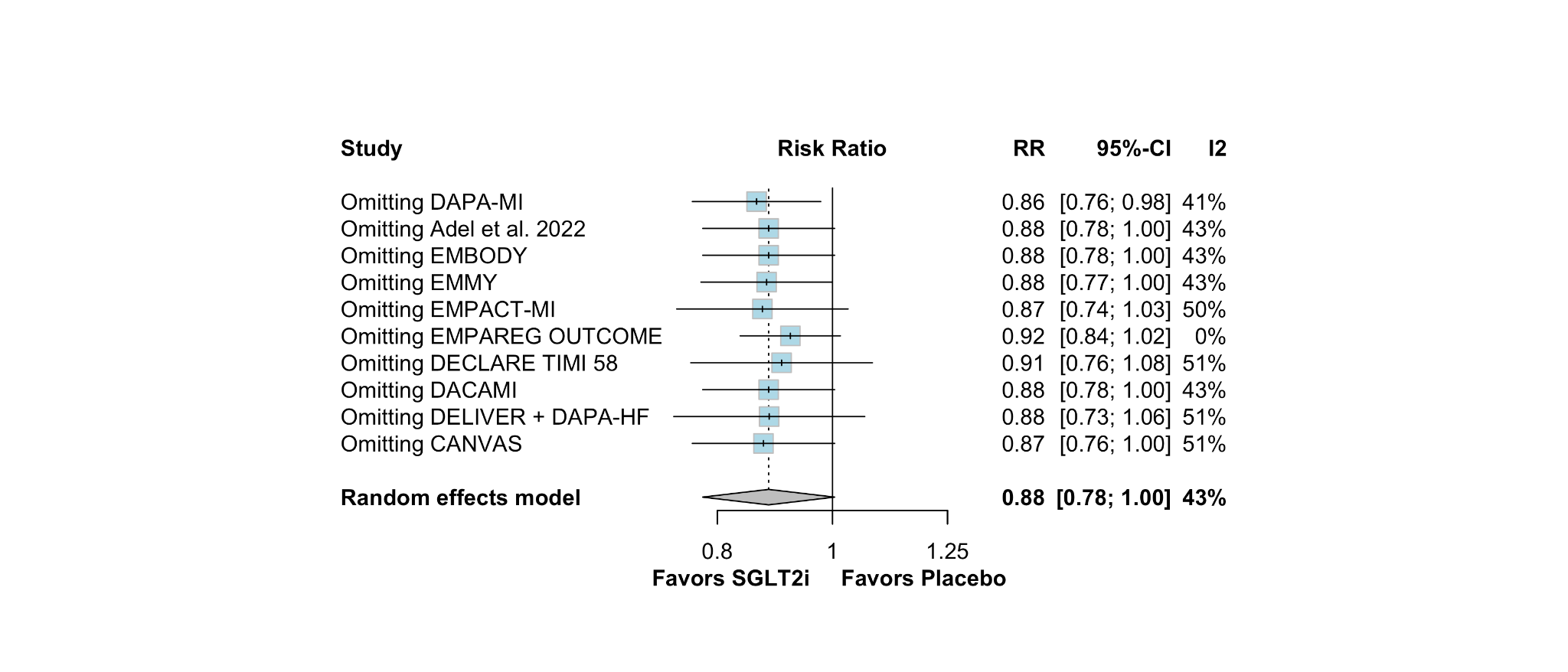


**Figure 8C.** Leave one out analysis for CV Death


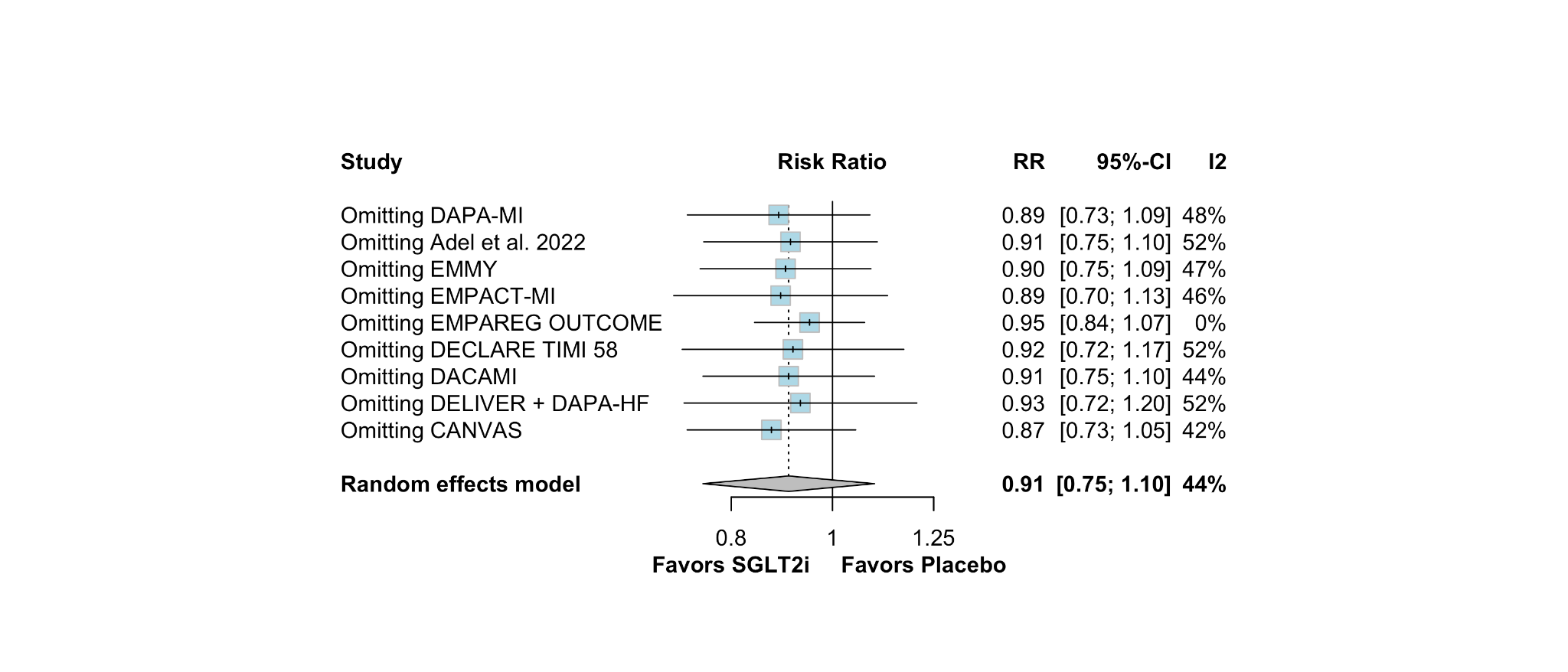


**Figure 8D.** Leave one out analysis for MI recurrence


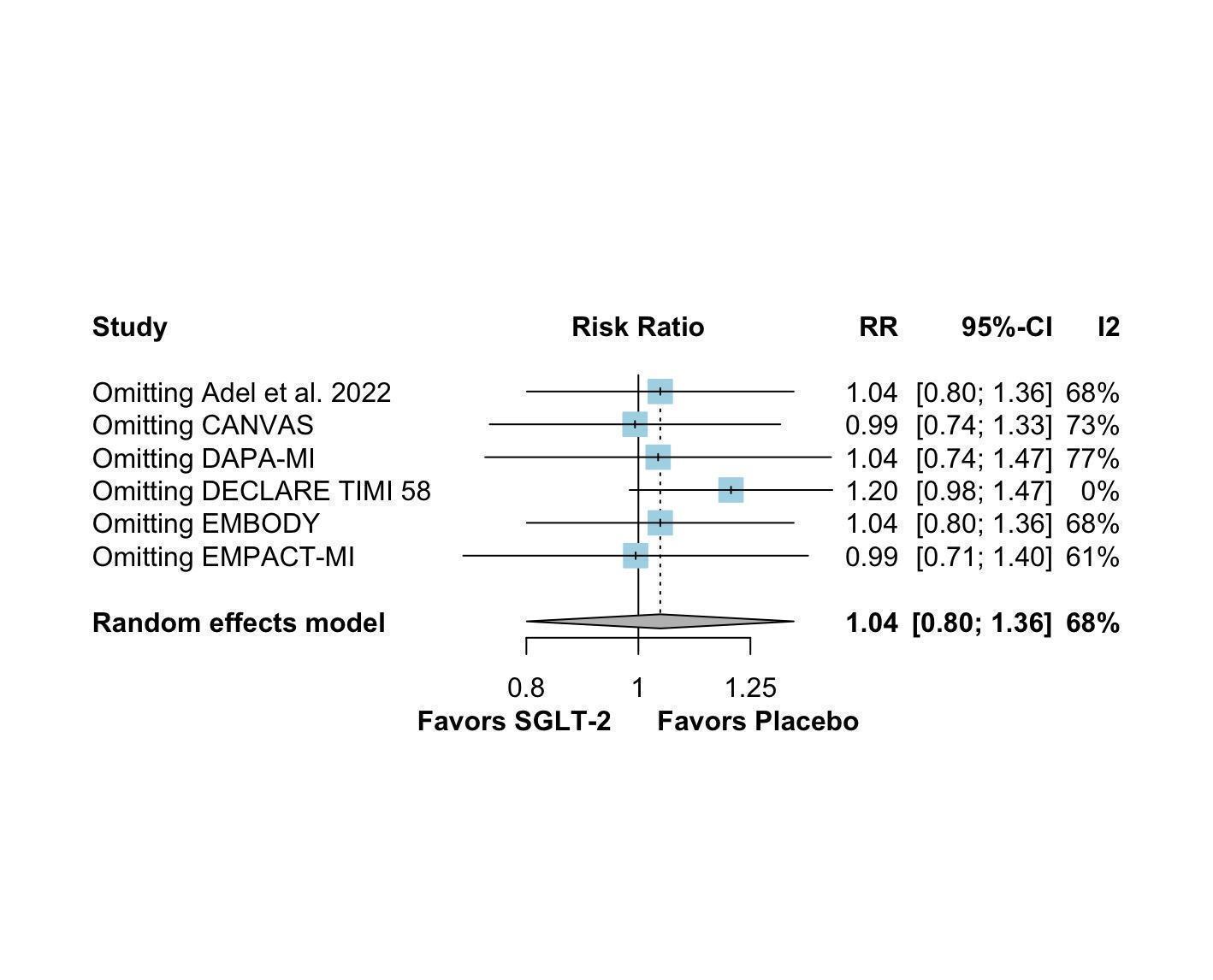


**Supplemental Figure 9. Post Hoc Sensitivity Analyses for the Primary Endpoint**
**Figure 9A**. Forest Plot for patients with LEVF<50% in acute MI setting


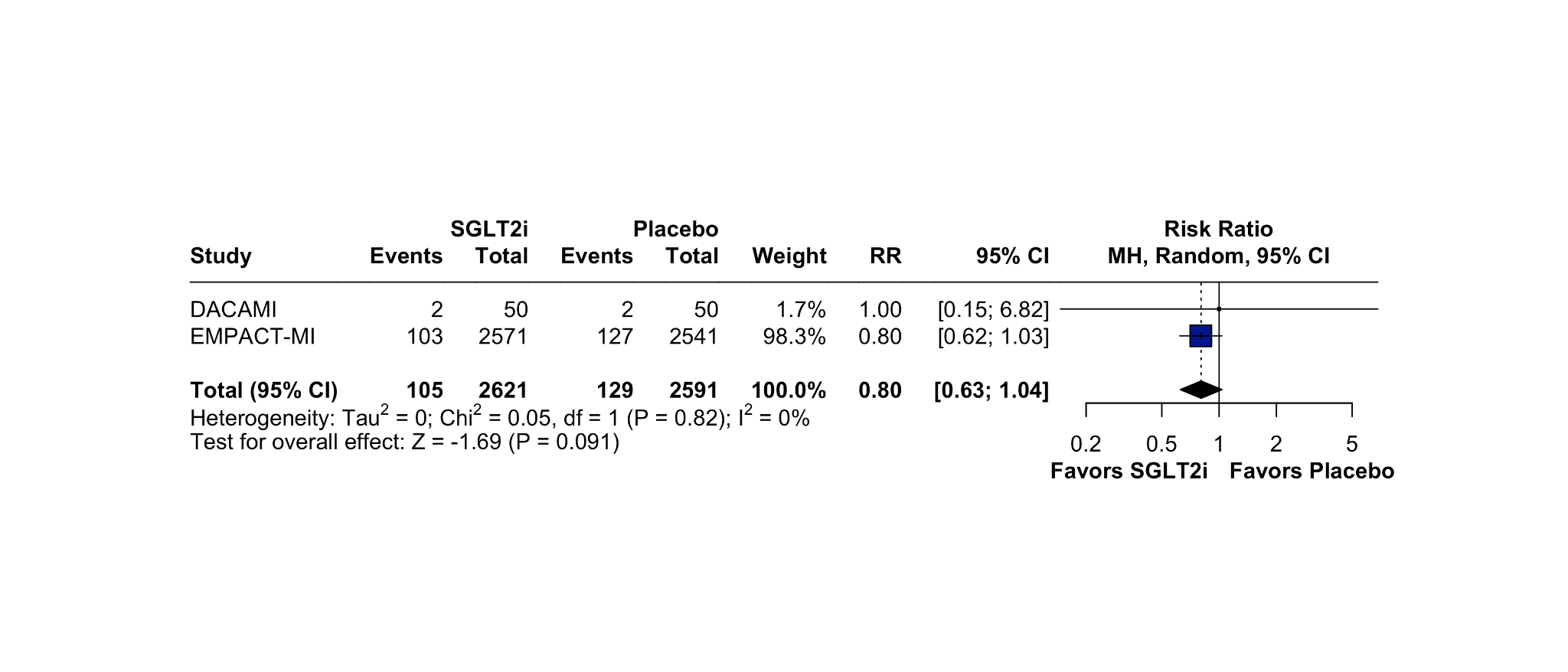


**Figure 9B**. Forest Plot for patients with STEMI in acute MI setting

**
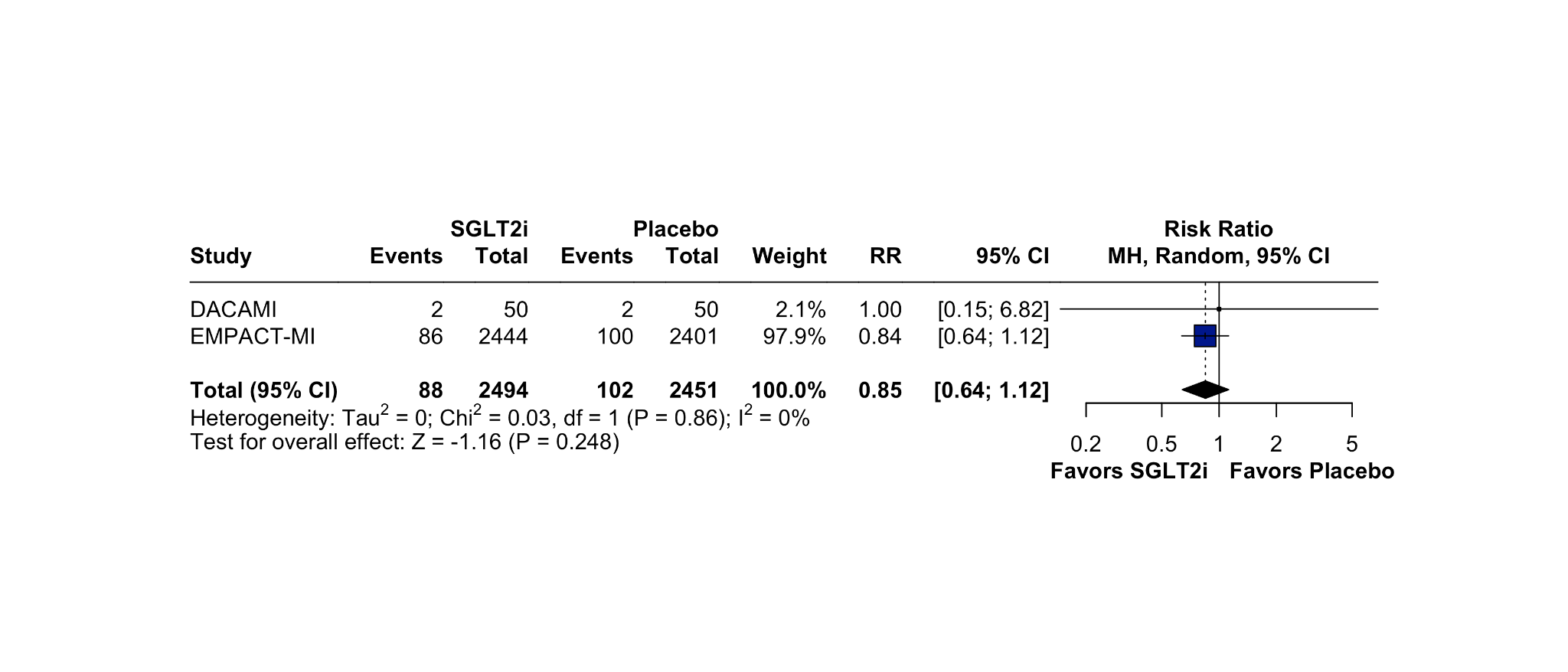
**

**Figure 9C.** Sensitivity Analysis after removing pre specified analysis or *post hoc* studies

**
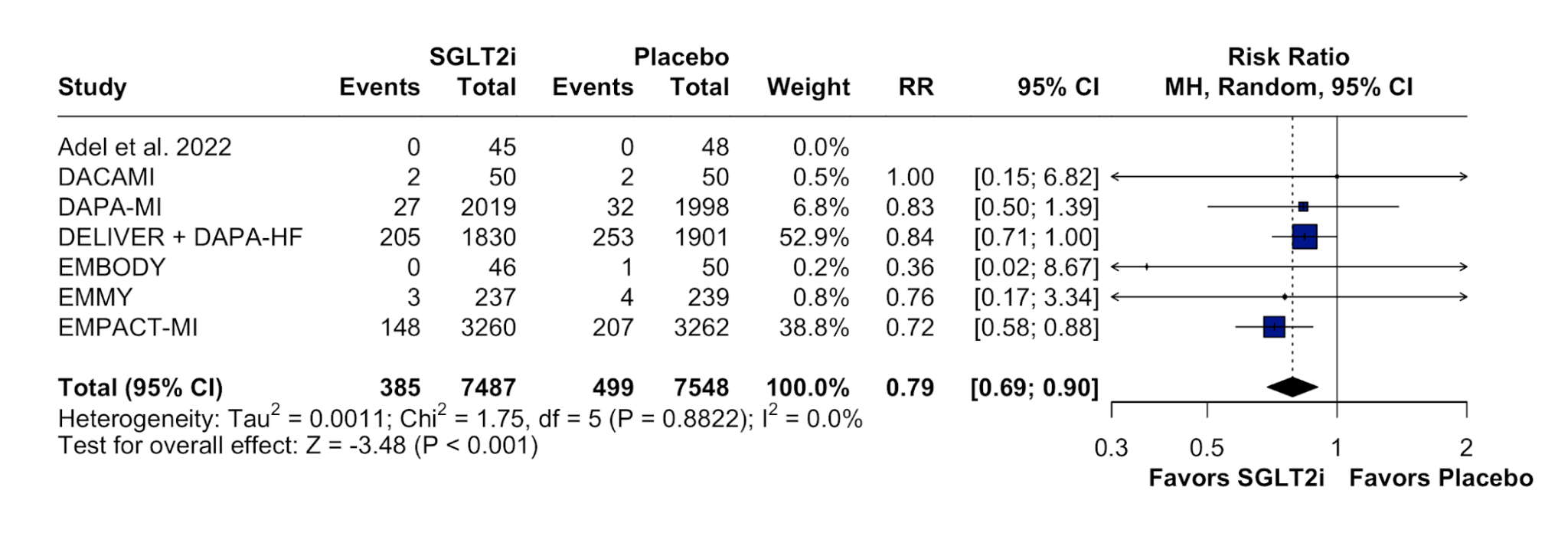
**

**Supplemental Figure 10. Trial Sequential Analysis**

**Figure 10A.** Trial Sequential Analysis for the primary endpoint


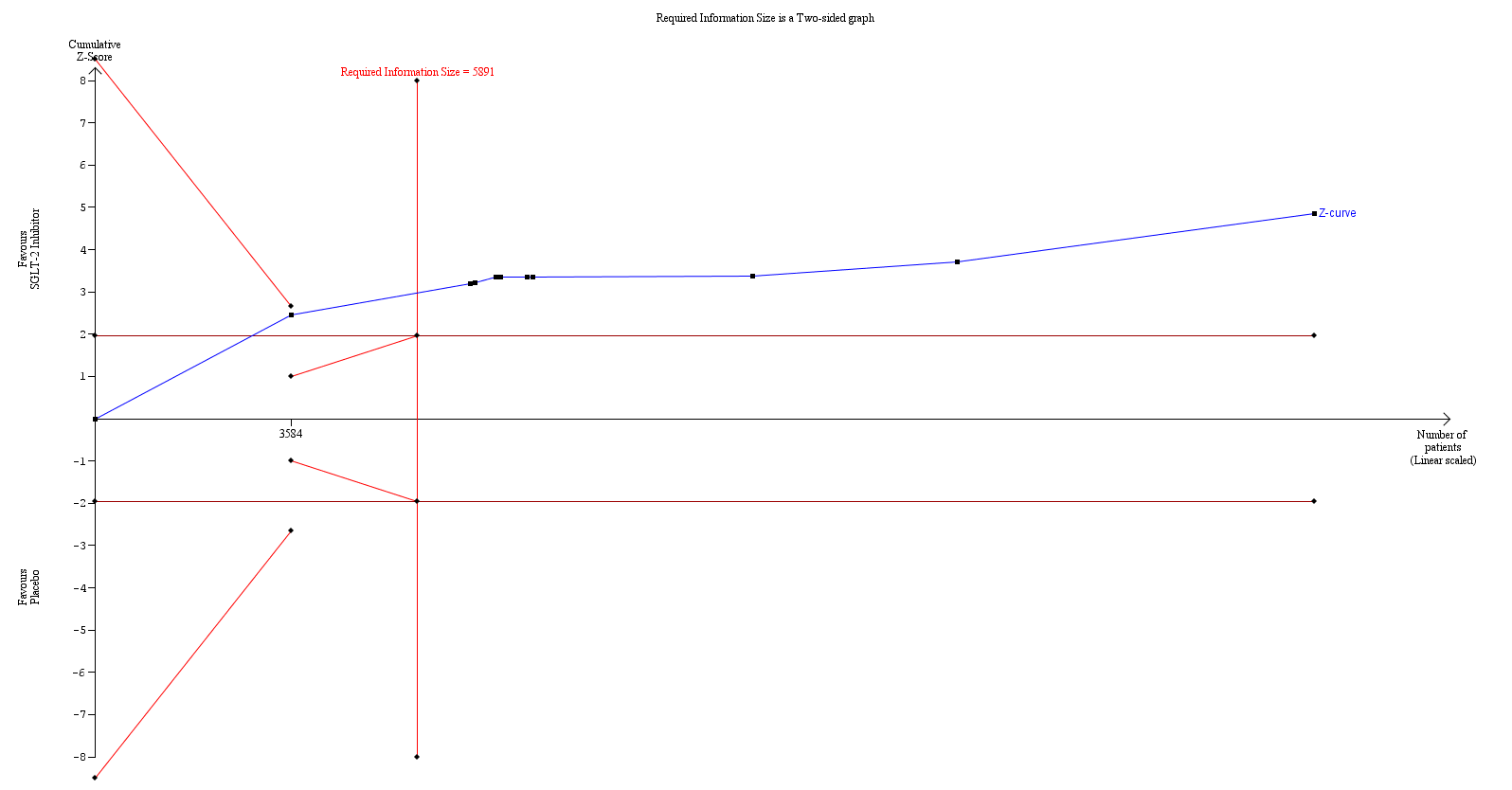


**Figure 10B.** Trial Sequential Analysis for the previous MI subgroup of the primary endpoint


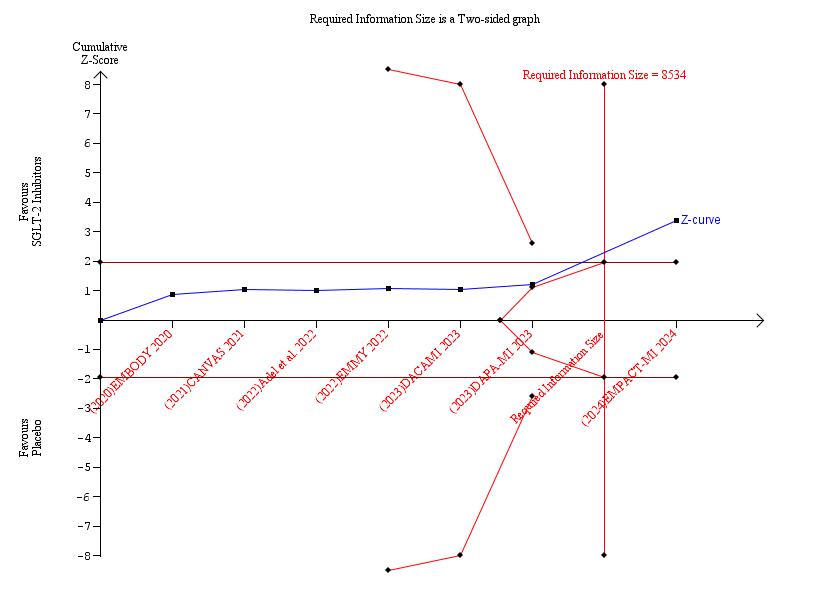


**Figure 10C.** Trial Sequential Analysis for the new onset MI subgroup of the primary endpoint


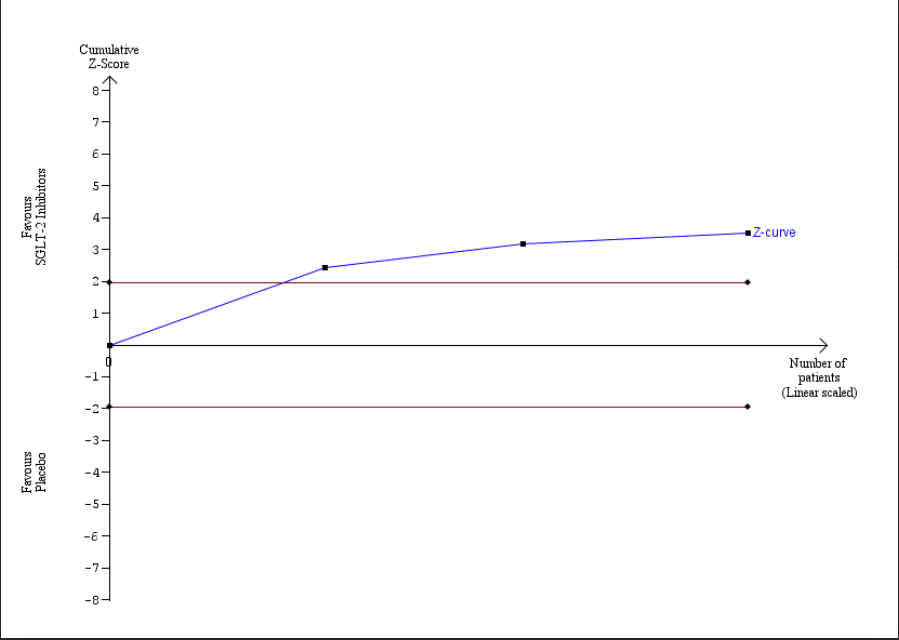


**Figure 10D.** Trial Sequential Analysis for the DM II subgroup of the primary endpoint


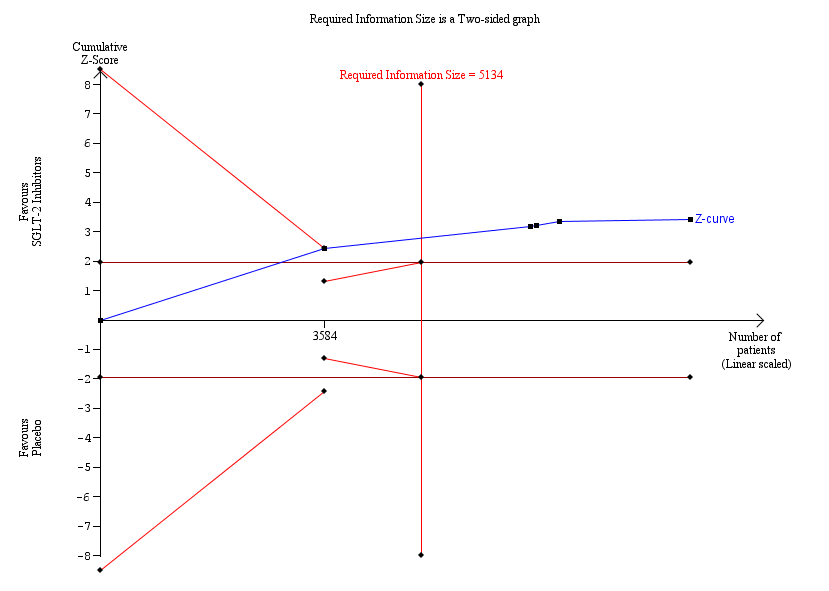


**Figure 10E.** Trial Sequential Analysis for the non-DM II subgroup of the primary endpoint


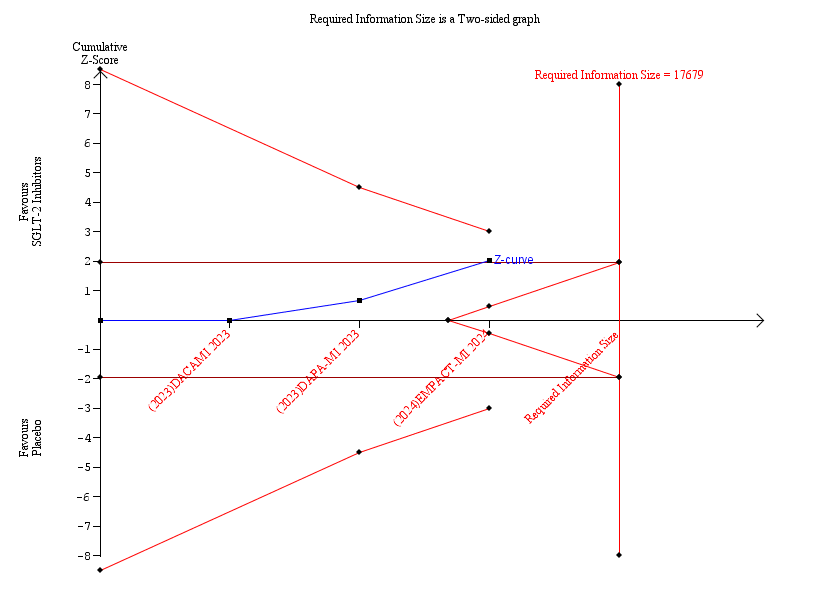


**Figure 10F.** Trial Sequential Analysis for the MACE Endpoint


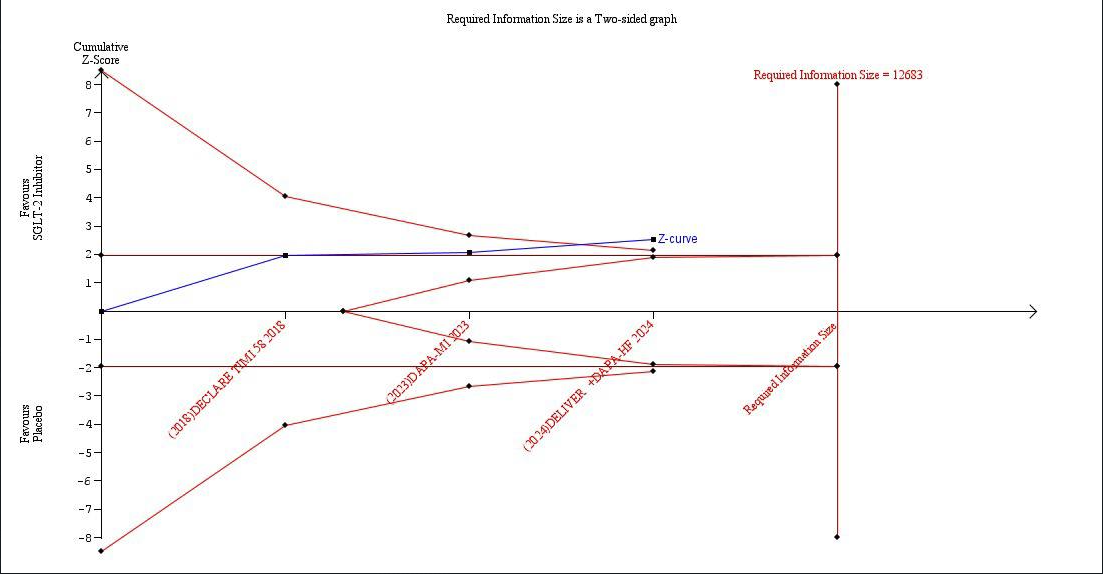


**Supplemental Figure 11. Risk of Bias 2 of All Included Studies**

**Figure 11A.** "Traffic light" plot of the domain-level judgments for each study


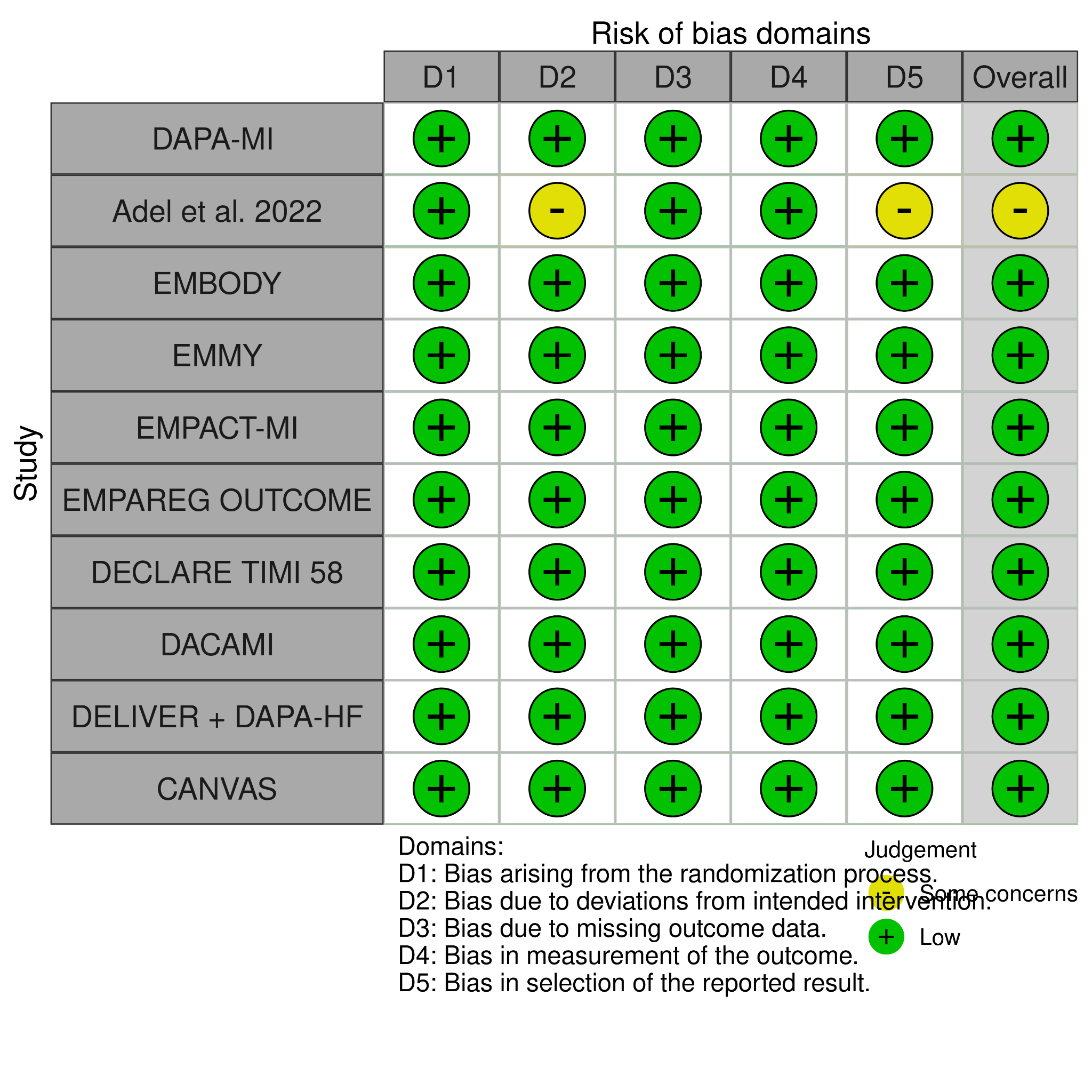


**Figure 11B.** Summary of overall weighted bar plot of risk-of-bias judgments within each bias domain.
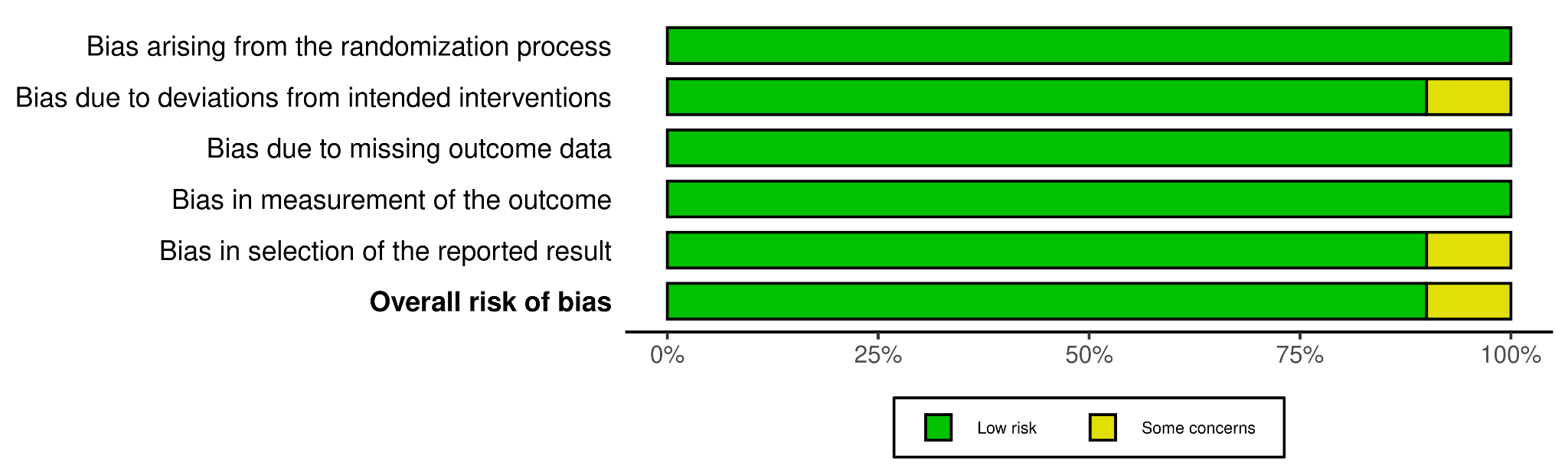


**Supplemental Figure 12. Funnel Plot and Egger’s Test for the Primary Efficacy Endpoint**

**
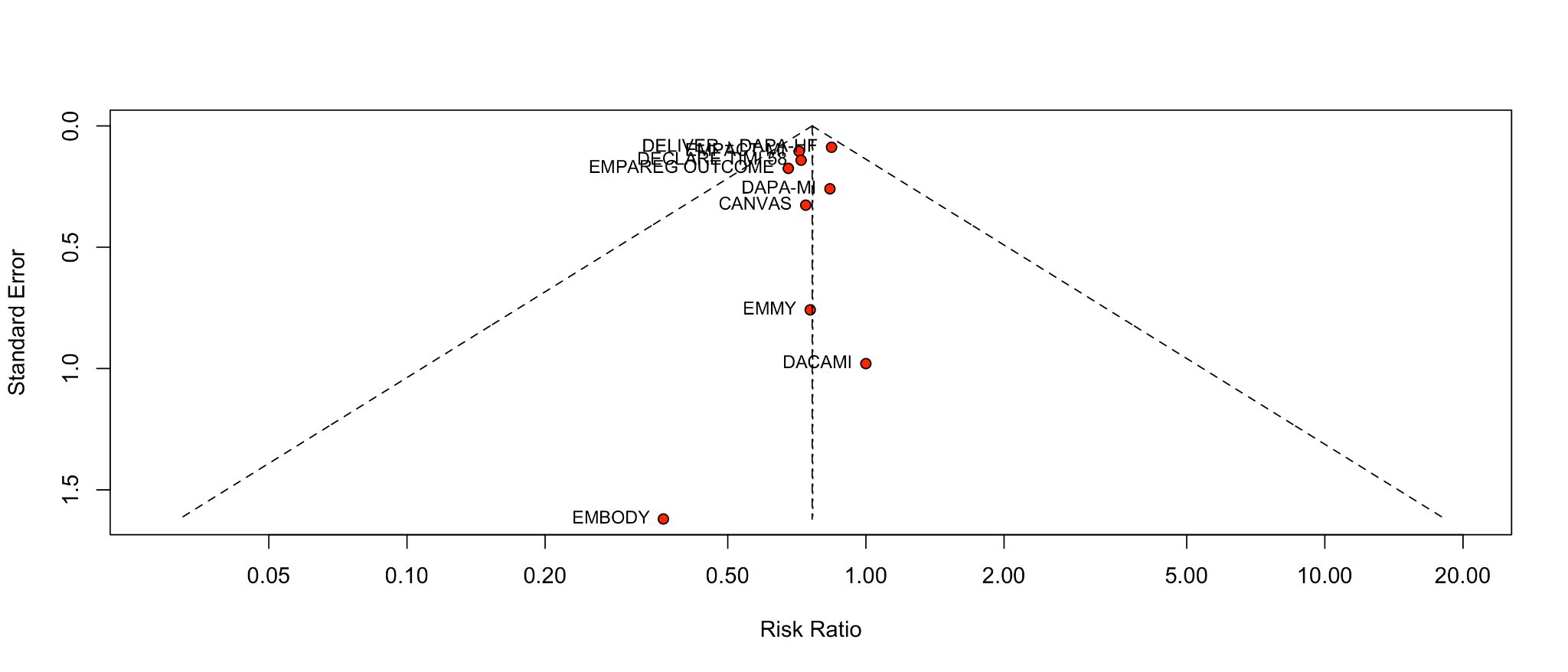
**

*Legend:* Funnel plot shows no asymmetry among the included studies, suggesting no publication bias

| **Egger’s Regression Test for the Primary Endpoint** | | | |
| --- | --- | --- | --- |
| **Intercept** | **95% CI** | **t** | **p-value** |
| -0.066 | -0.6 to -0.47 | -0.241 | 0.8159319 |

**Supplemental References**

1. Agarwal MA, Fonarow GC, Ziaeian B. National Trends in Heart Failure Hospitalizations and Readmissions From 2010 to 2017. JAMA Cardiol 2021;6:952. https://doi.org/10.1001/jamacardio.2020.7472.

2. National Center for Chronic Disease Prevention and Health Promotion (US) Office on Smoking and Health. The Health Consequences of Smoking—50 Years of Progress: A Report of the Surgeon General. Atlanta (GA): Centers for Disease Control and Prevention (US); 2014. 11, General Morbidity and All-Cause Mortality. Available from: https://www.ncbi.nlm.nih.gov/books/NBK294300/ n.d.

3. Hicks KA., Mahaffey KW., Mehran R., et al. 2017 Cardiovascular and Stroke Endpoint Definitions for Clinical Trials. J Am Coll Cardiol 2018;71(9):1021–34. Doi: 10.1016/j.jacc.2017.12.048.

4. Shimizu W., Kubota Y., Hoshika Y., et al. Effects of empagliflozin versus placebo on cardiac sympathetic activity in acute myocardial infarction patients with type 2 diabetes mellitus: the EMBODY trial. Cardiovasc Diabetol 2020;19(1):148. Doi: 10.1186/s12933-020-01127-z.

5. Zinman B., Wanner C., Lachin JM., et al. Empagliflozin, Cardiovascular Outcomes, and Mortality in Type 2 Diabetes. New England Journal of Medicine 2015;373(22):2117–28. Doi: 10.1056/NEJMoa1504720.

6. Adel SMH., Jorfi F., Mombeini H., Rashidi H., Fazeli S. Effect of a low dose of empagliflozin on short-term outcomes in type 2 diabetics with acute coronary syndrome after percutaneous coronary intervention. Saudi Med J 2022;43(5):458–64. Doi: 10.15537/smj.2022.43.5.20220018.

7. Butler J., Jones WS., Udell JA., et al. Empagliflozin after Acute Myocardial Infarction. New England Journal of Medicine 2024;390(16):1455–66. Doi: 10.1056/NEJMoa2314051.

8. Dayem KA., Younis O., Zarif B., Attia S., AbdelSalam A. Impact of dapagliflozin on cardiac function following anterior myocardial infarction in non-diabetic patients – DACAMI (a randomized controlled clinical trial). Int J Cardiol 2023;379:9–14. Doi: 10.1016/j.ijcard.2023.03.002.

9. James S., Erlinge D., Storey RF., et al. Dapagliflozin in Myocardial Infarction without Diabetes or Heart Failure. NEJM Evidence 2024;3(2):EVIDoa2300286. Doi: 10.1056/EVIDoa2300286.

10. Yu J., Li J., Leaver PJ., et al. Effects of canagliflozin on myocardial infarction: a post hoc analysis of the CANVAS programme and CREDENCE trial. Cardiovasc Res 2022;118(4):1103–14. Doi: 10.1093/cvr/cvab128.

11. Fitchett D., Inzucchi SE., Cannon CP., et al. Empagliflozin Reduced Mortality and Hospitalization for Heart Failure Across the Spectrum of Cardiovascular Risk in the EMPA-REG OUTCOME Trial. Circulation 2019;139(11):1384–95. Doi: 10.1161/CIRCULATIONAHA.118.037778.

12. von Lewinski D., Kolesnik E., Tripolt NJ., et al. Empagliflozin in acute myocardial infarction: the EMMY trial. Eur Heart J 2022;43(41):4421–32. Doi: 10.1093/eurheartj/ehac494.

13. Peikert A., Vaduganathan M., Claggett BL., et al. Dapagliflozin in patients with heart failure and previous myocardial infarction: A participant‐level pooled analysis of <scp>DAPA‐HF</scp> and <scp>DELIVER</scp>. Eur J Heart Fail 2024;26(4):912–24. Doi: 10.1002/ejhf.3184.
